# Laccase-mediated synthesis of bioactive natural products and their analogues

**DOI:** 10.1039/d1cb00259g

**Published:** 2022-04-18

**Authors:** Nunzio Cardullo, Vera Muccilli, Corrado Tringali

**Affiliations:** Dipartimento di Scienze Chimiche, Università degli Studi di Catania V.le A. Doria 6 95125-Catania Italy v.muccilli@unict.it ctringali@unict.it +39-095-580138 +39-095-7385041 +39-095-7385025

## Abstract

Laccases are a class of multicopper oxidases that catalyse the one-electron oxidation of four equivalents of a reducing substrate, with the concomitant four-electron reduction of dioxygen to water. Typically, they catalyse many anabolic reactions, in which mostly phenolic metabolites were subjected to oxidative coupling. Alternatively, laccases catalyse the degradation or modification of biopolymers like lignin in catabolic processes. In recent years, laccases have proved valuable and green biocatalysts for synthesising compounds with therapeutic value, including antitumor, antibiotic, antimicrobial, and antioxidant agents. Further up to date applications include oxidative depolymerisation of lignin to gain new biomaterials and bioremediation processes of industrial waste. This review summarizes selected examples from the last decade's literature about the laccase-mediated synthesis of biologically active natural products and their analogues; these will include lignans and neolignans, dimeric stilbenoids, biflavonoids, biaryls and other compounds of potential interest for the pharmaceutical industry. In addition, a short section about applications of laccases in natural polymer modification has been included.

## Abstract 2

Laccases are green biocatalysts to synthesise compounds with therapeutic value.

## Graphical abstract

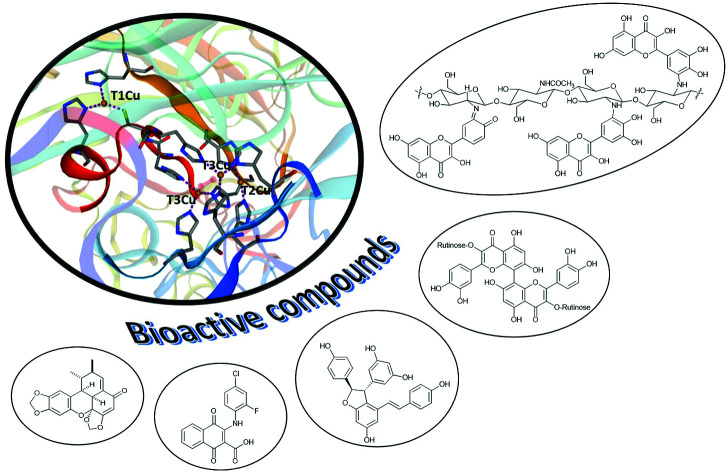

## 1. Introduction

**Fig. 1:**
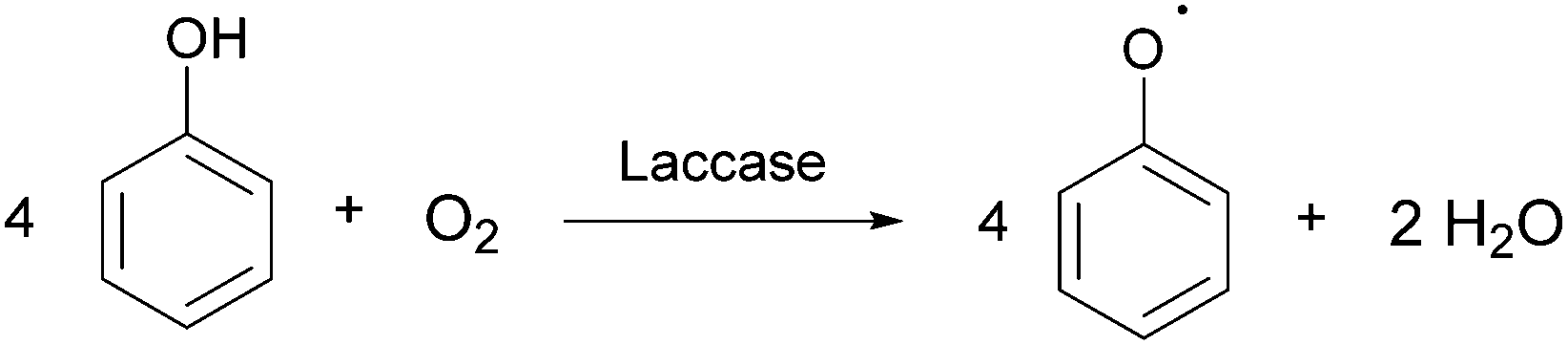
The reaction catalysed by laccase of a representative phenolic substrate.

Laccases (EC 1.10.3.2 *p*-diphenol oxidoreductase, or multicopper oxidase) are a group of oxidative enzymes widespread in plants, bacteria, fungi, and animal kingdom, showing a broad substrate specificity and working without the need of cofactors.^1^ These enzymes oxidise aromatic substrates, mainly phenolics but also diamines or benzenethiols, simply through the reduction of molecular oxygen into water, which is the only by-product (Fig. 1).^2^ This reaction occurs in the presence of four copper ions involved in electron transfer from the substrate to oxygen, which is the final electron acceptor. In the last two decades, a significant increase in exploitation of these biocatalysts in organic synthesis as well as in pharmaceutical, agrochemical, food processing, and industrial textile processes^3^ was observed; this is also related to the development of green chemistry, based on sustainable, eco-friendly, cost-effective, and possibly high yielding methods.^4^

The first laccase was isolated in *Rhus vernicifera* from the Japanese lacquer tree *Toxicodendron vernicifluum* in 1883; since then, laccases have been identified in several higher plant species.^5^ However, these enzymes have been found mainly in fungi: typical laccase producers are the wood-decaying fungi that employ these enzymes for lignin degradation.^6^ Moreover, laccases also occur in insects where they are involved in the sclerotization of cuticles and probably in their immune response;^7^ they have also been identified in more than 20 bacterial species.^8^

**Fig. 2:**
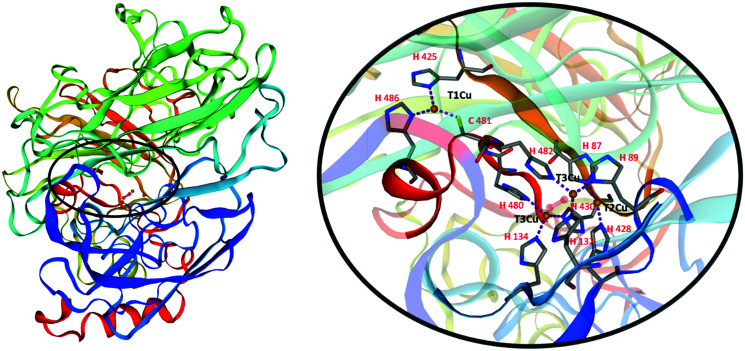
3D structure of *Trametes versicolor* laccase (White-rot fungus; *Coriolus versicolor*; uniprot Q12717). Homology model based on 2xyb.1.A, SMTL Version 2021-07-14; Seq Identity 72.78%. “Crystal structure of a fully functional laccase from the ligninolytic fungus *Pycnoporus cinnabarinus*”. Details on the active site of laccase show the internal transfer pathway from the T1Cu site to the T2Cu/T3Cu trinuclear cluster, and the amino acid residues coordinating Cu^2+^ ions.

Laccases, also called blue-copper oxidases, as one of the copper atoms, the paramagnetic type 1, (T1Cu), is responsible for their characteristic colour. Different multicopper oxidases are structurally similar. The analysis of the three-dimensional structure of laccases (Fig. 2) has revealed two distinct active sites involved in the reaction mechanism: a mononuclear T1Cu site where the one-electron oxidation of four equivalents of a given substrate occurs to yield organic radicals, and a trinuclear cluster T2Cu/T3aCu/T3bCu where molecular oxygen is reduced to two water molecules (Fig. 2).^9^

The T1Cu is coordinated with a trigonal planar geometry to the Nδ1 of two histidine residues (H425 and H486 in Fig. 2) and to the sulphur atom of a cysteine (C481 in Fig. 2) in laccases active site. The reduction step, occurring to the T1Cu site is the rate-limiting step of the catalytic process. Once accepted, the electrons are transferred through the highly conserved His–Cys–His tripeptide to the trinuclear cluster to reduce O_2_ in water.

The type of axial ligand, the coordination sphere, and solvent accessibility to the T1Cu influence the redox potential *E*^0^. Differently from low-redox-potential enzymes, mainly present in bacteria, plants and insects (*E*^0^ = 340–490 mV), which limits their use to phenolic substrates; fungal laccase possesses higher redox potential at the T1 site (*E*^0^ = 500–800 mV) and can work with a larger variety of substrates.^10^ It is widely accepted that the high redox potential of most fungal laccases arises from the lack of the methionine, involved in a long axial bond, in stabilising the T1Cu trigonal planar complex. Moreover, the low-potential laccases have a methionine as a non-coordinating axial ligand, whereas the high-potential laccases possess a phenylalanine as a non-coordinating axial ligand.^9^ Furthermore, according to other findings, the coordination distances between Cu and the two Nδ1H are correlated with the redox potential of T1Cu and thus, the longer the distances, the higher the Cu electron deficiency, the higher the redox potential.

Both plant and fungal laccases are glycosylated enzymes with plant enzymes bearing higher extent of glycosylation (22–45%) than the fungal ones (10–25%) and consequent higher molecular masses on SDS–PAGE. The glycosylation is useful for the copper retention, secretion, thermal stability and enzymatic activity.^11^ In all laccases (bacterial, fungal and plant), some differences at catalytic site have been reported, corresponding to differences in functional diversity and evolutionary relationship.^12^ The fungal laccases usually show lower thermal stability than bacterial ones.^13^ The thermal stability probably arises from the interaction between the copper ions and salt bridges or hydrogen bonding network.^14^ Bacterial laccases are highly active and much more stable at harsh condition such as high pH, high temperatures, and high concentrations of copper ions and chloride.^12^ For the above reasons, the immobilized bacterial laccases are more suitable for almost all industrial processes.^12^

**Fig. 3:**
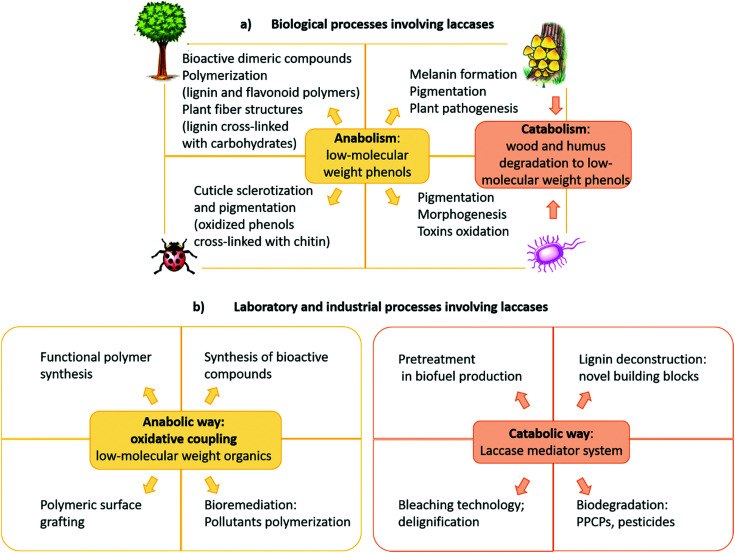
(a) Biological, (b) laboratory and industrial processes involving laccases. PPCPs: pharmaceuticals and personal care products.

The role of laccases in Nature varies with the organism and they participate in both anabolic and catabolic processes (Fig. 3a).^15^ In typical anabolic reactions,^16^ low molecular weight phenolics (monolignols and flavonoids) are oxidized to radical and/or quinone intermediates, which react to yield several types of dimeric products, thus providing in many cases compounds with biological activity. Because these dimers still contain phenolic functions, they can form dimeric radicals, thus producing trimers, oligomers, and polymers by self/cross-coupling.^17^ Thus, laccases promote the biosynthesis of a variety of dimers including lignans and related compounds,^1,5,10,18–20^ as well as of polymeric products such as lignin, flavonoid polymers, melanins,^21^ quinones cross-linked to cuticular proteins (for cuticle sclerotization in insects),^22^*etc.* Laccase-mediated catabolic processes include lignin/lignocellulose depolymerisation and humus degradation^23^ and may represent a powerful biotechnological tool in a number of industrial applications.

Most importantly, laccase-mediated reactions are attractive in organic chemistry for the laboratory synthesis of fine chemicals and biologically active compounds as well as for industrial applications (Fig. 3b). Namely, these enzymes have proved to be useful biocatalysts in the ecofriendly synthesis of bioactive compounds,^24,25^ including antitumor,^26–28^ antibiotic,^17^ antimicrobial,^29^ and antioxidant agents.^30^ Furthermore, laccases have been employed to prepare new polymeric biomaterials,^31,32^ such as hair dyes and skin lightening agents,^33^ as well as in the pollutant polymerization.^34^ Laccases are also employed in biorefineries to produce biofuels by degradation of lignocellulosic biomasses in the pulp and paper industry,^35,36^ in the production of chemical building blocks from biomass-based materials and in the biodegradation of pesticides, pharmaceuticals and personal-care products (PPCPs).^37–39^ These synthetic applications have highlighted that pH, solvent and chemical mediators can improve the enzyme efficiency, thus enabling laccases to oxidize a wider range of substrates in addition to phenolic compounds. Moreover, a wide number of supports, either inorganic, organic, or hybrids, have been employed to immobilize the enzyme, to overcome some practical limitations,^34,40^ namely to increase the surface/volume ratio, thus avoiding the use of large amounts of enzyme for large-scale synthesis and allowing its effective recycling. Further advantages are the enhanced catalytic activity and stability of supported system respect to the free laccase, as well as the extension of the applied temperature and pH ranges. Laccase immobilization can be achieved by adsorption or covalently binding onto supporting materials, or by less traditional and less expensive methods such as membranes or gels encapsulation.^41^ Several types of nanocarriers have been developed. For instance, *Trametes versicolor* laccase (TvL) immobilized onto silica-based nanoparticles, showed excellent stability by retaining about 80% of its initial activity after 15 reaction cycles in a one-pot synthesis of a set of chromene derivatives.^42^ Employment of nanomaterials, including nanocarbon materials, nanomembranes and nanocomposites with large specific surface area and high binding energy, improves the enzyme loading amount compared with other immobilization methodologies.^43^ Immobilized laccases are employed in water purification, as well as in micro-pollutant and phenolic compounds removal. Recent works focused on the oxidation of model compounds by TvL-immobilised onto wood by golden nanoparticle-mediated adsorption. The results pointed out remarkable stability of the wood–gold–enzyme hybrid retaining about 90% of its starting activity after 25 cycles of oxidation, thus making the system a valuable heterogeneous biocatalyst for continuous-flow processes in chemical industry.^44^

Indeed, since the beginning of the 21st century, laccases have been extensively engineered to develop enzymes with higher activity and stability more suitable for large-scale biotechnological applications in various industries.^3^

A number of excellent reviews, including laccase-mediated synthesis of natural or bioinspired products, have been published in recent years.^17,24,45,46^ This review aims to highlight selected examples from the last decade literature about the laccase-mediated synthesis of biologically active natural products and their analogues; this will include the most representative classes of dimeric products: lignans and neolignans, dimeric stilbenoids, biflavonoids, biaryl and other compounds of potential interest for the pharmaceutical industry. In addition, a short section about applications of laccases in natural polymer modification has been included.

## 2. General schemes of laccase-mediated oxidative coupling

Biosynthetic oxidative coupling of small monomeric molecules readily generates homo- and heterodimers with high chemical diversity and structural complexity, frequently displaying significant bioactivity.^47^ In addition, dimeric compounds often show stronger activity than their corresponding parent monomers.^48^ Some of these compounds have become therapeutic agents or have promising biological properties. Examples are 3′,4-di-*O*-methylcedrusin (1), an antiproliferative compound which inspired the synthesis of analogues with antitumor properties;^49^ podophyllotoxin (2), a natural antimitotic whose optimization led to the anticancer drug etoposide;^50^ hypericin (3), a natural anthraquinone pigment utilized in the treatment of different types of cancer and with antimicrobial activity;^51^ gossypol (4) a potential therapeutic agent for resistant tumours and chronic human diseases.^52^
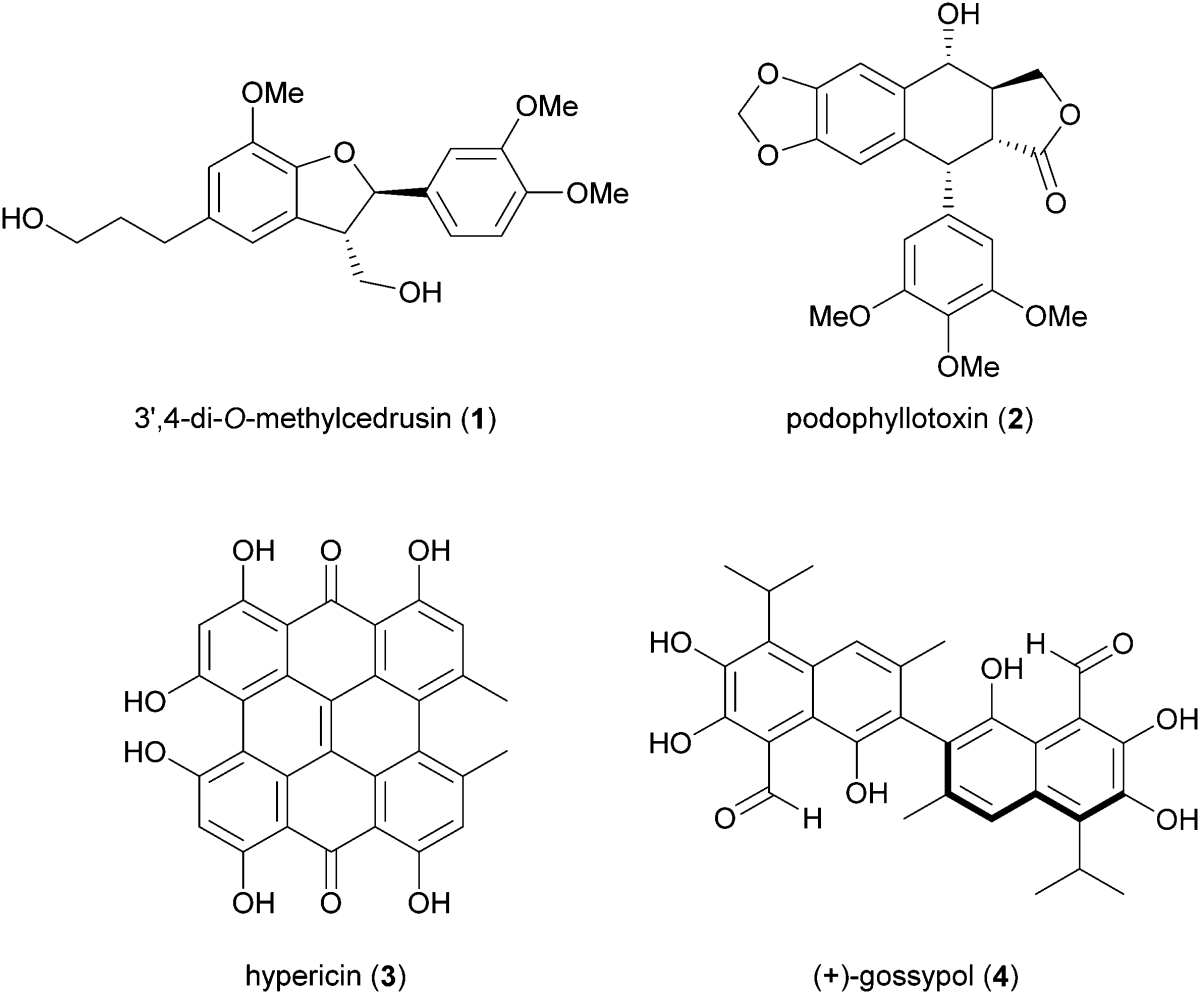


**Scheme 1:**
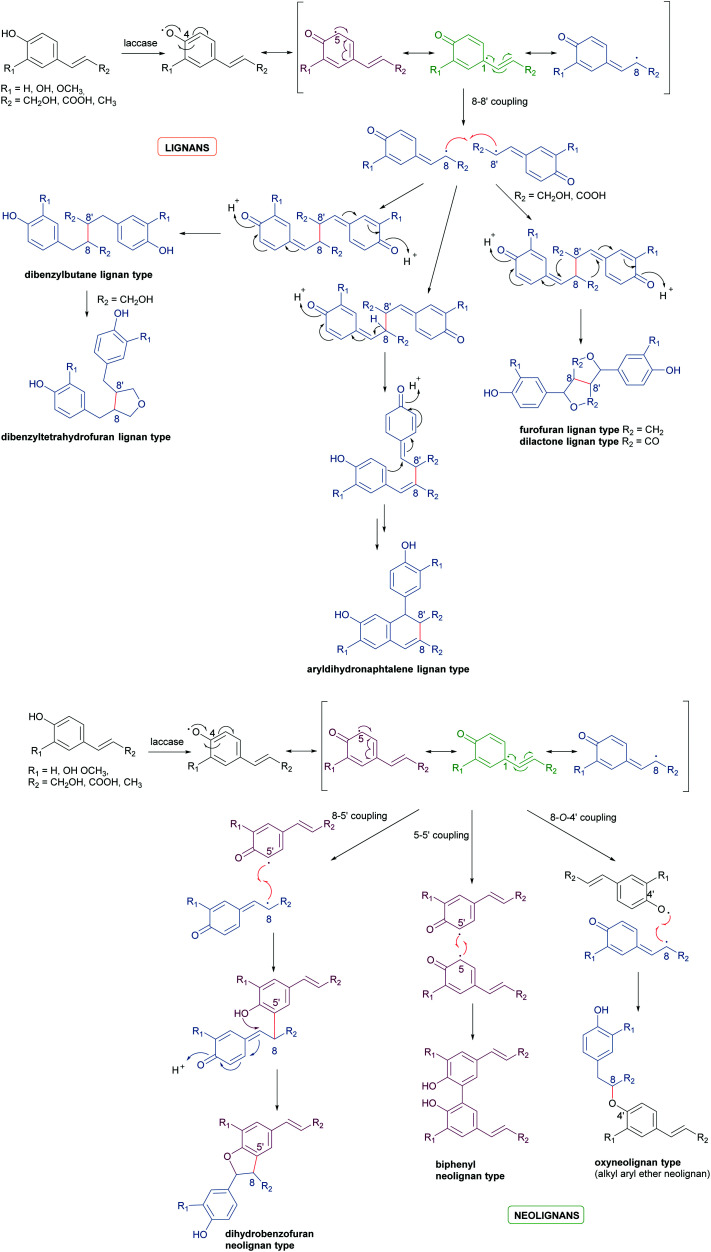
(a) Schematic representation of phenylpropanoids dimerization to give lignans. (b) Schematic representation of phenylpropanoids dimerization to give neolignans.

Biosynthetic oxidative coupling is usually a two-step process. In the case of phenolic compounds with a C6C3 skeleton (phenylpropanoids), laccases firstly give rise to radicals typically stabilised through mesomeric forms, as illustrated in Schemes 1a and b (oxidative step). Thus, in conjugated systems, including an aromatic moiety, the lone electron can also delocalize along with all the carbonaceous skeleton, thus making several positions reactive. In the further reaction (coupling step), two radicals join through the formation of a new C–C or C–O bond (Schemes 1a and b), affording a quinone intermediate which can undergo intramolecular reactions (cyclization, rearrangements) to form a variety of structurally different products. They can be generically called dimers; however, according to IUPAC, the products formed by coupling involving 8–8′ positions of phenylpropanoid radicals are called lignans, whereas those generated by coupling at positions different from 8–8′ are called neolignans; in particular, oxyneolignans show C6C3 units linked by an ether function.^47,53^ Other coupling products involving monomers different from phenylpropanoids have specific names, such as dimeric stilbenoids, dimeric flavonoids, *etc.*

**Scheme 2:**
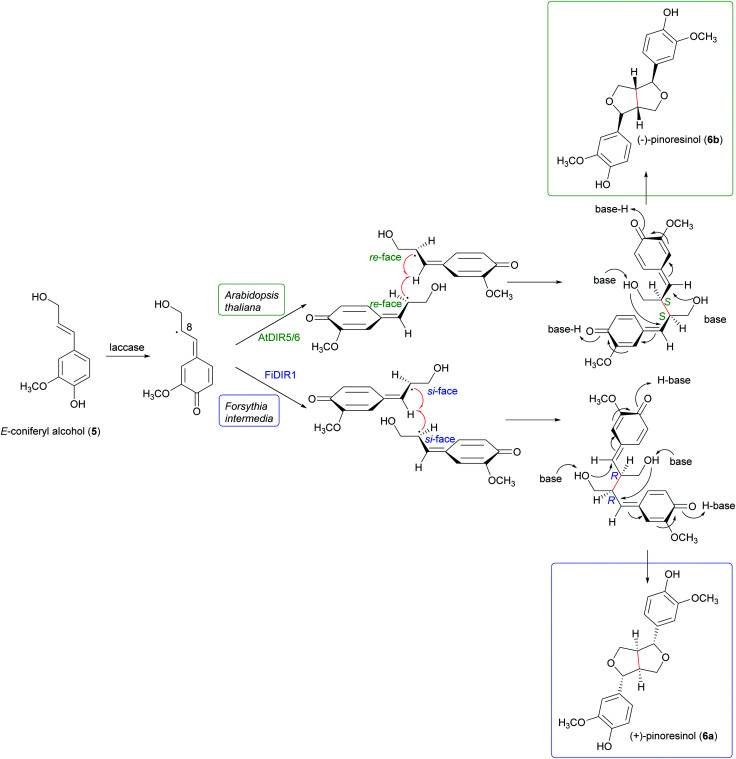
Biosynthetic formation of (+) and (−)-pinoresinol in plants. (+)-pinoresinol is biosynthesised in *Forsythia intermedia* by FiDIR1; conversely, (−)-pinoresinol is biosynthesised in *Arabidopsis thaliana* by AtDIR5/6.^58^

Schemes 1a and b highlight the variety of compounds that can be biosynthesized through oxidative coupling; many of these possess one or more stereogenic centres and are produced with high stereoselectivity.^54^ According to the most recent findings, some oxidizing enzymes are able to control the regio- and stereochemistry of radical coupling.^55^ However, most of the above-cited biosynthetic processes requires two components: an oxidizing agent to generate radicals and a “dirigent protein” (DIR)^56^ that leads, properly orienting the two radicals, to regio- and stereoselective coupling, as in the reported work about the biosynthesis of (+)-pinoresinol (Scheme 2).^57^ To date, the DIR-mediated coupling control has been studied only for the biosynthesis of some types of lignans,^58–60^ the stilbenoid dimer (+)-ε-viniferin,^61^ and ellagitannins such as tellimagrandin II and cornusiin E.^62–64^ Nevertheless, the large number of DIR with unknown functions in plants and the prevalence of enantiopure natural products in the plant kingdom, provides the foundation for discovering novel DIR-mediated reactions.

**Scheme 3:**
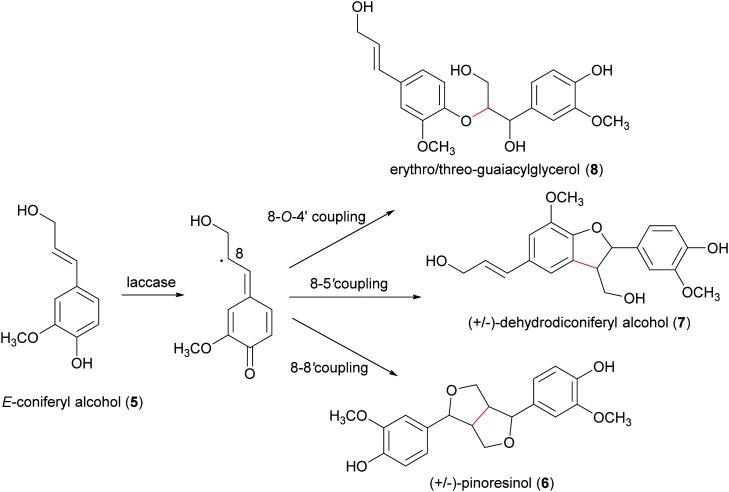
Bimolecular phenoxy radical coupling products from *E*-coniferyl alcohol.

Inspired by these biosynthetic reactions, many researchers carried out laboratory-scale biomimetic syntheses to obtain natural dimeric compounds or their analogues, employing commercially available oxidases, namely laccases and peroxidases.^65–69^ Most peroxidases are ferric-heme proteins catalysing the one electron oxidation of a substrate in the presence of hydrogen peroxide, which is reduced to water. A less environmentally friendly methodology is based on the use of oxidizing agents such as Ag_2_O, FeCl_3_, MnO_2_ and others.^70–75^ Both methods mimic the biosynthetic pathway, as the key step is based on the oxidative coupling of radicals. If regioselectivity during radical coupling may be in part controlled by thermodynamically favoured mesomeric structures or by steric hindrance effects, enantioselectivity is not energetically discriminated. Thus, laboratory reactions afford racemic mixtures and often also a mixture of isomeric products with different structures (Scheme 3).

Several parameters can affect laccases performance and stability, among these pH, temperature and solvents; in addition, different laccases can afford different dimeric products or the same mixture of products with different relative ratios.^76^ Substrate specificity and affinity of laccase vary with changes in pH. Usually the most common buffers employed in fungal and bacterial laccase-mediated reactions are phosphate and acetate, with pH ranging from neutral (7.0) to weakly acid (4.5)^76^ and may increase to pH 9 for plant laccases. The pH-activity relationship seems to be linked to the physiological functions of laccases, rather than the type of substrate.^12^ In addition, the optimal temperature ranges from 50 °C to 70 °C but a few enzymes have been employed at temperature below 35 °C.^12^ Very often these reactions can be carried out in organic solvents, useful for dissolving hydrophobic substrates, without significantly decreasing in laccase activity. Several examples include the use of water-miscible organic co-solvents but also water–organic biphasic systems.^27^ Of note, in some cases, the solvent can exert stabilizing effects of a specific radical, thus promoting the formation of a specific product with high selectivity.^45^ Ionic liquids represent an alternative to common molecular organic solvents, having many advantages such as tuneable hydrophobicity and water-miscibility, high solubility of enzymes, negligible vapour pressure and good thermal stability.^77,78^ A number of researches have exploited the effect of various ionic liquids on laccases using the oxidation of 2,2-azinobis-(3-ethylbenzothiazoline-6-sufonic acid) (ABTS) as a model reaction. Many results show that ionic liquids reduce the substrate selectivity but enhance the laccase reactivity. However, higher amounts of ionic liquids can inhibit the laccase activity.^77^ Additionally, some ionic liquids are not strictly “green” and may be even more toxic than some organic solvents. More recent results have identified natural deep eutectic solvents among ionic liquids as valuable green solvents for laccase-catalysed biotransformations.^79,80^ In particular, betaine-based eutectics showed the best features for laccase stability at higher temperatures, thus enhancing the enzyme effectiveness.^79^

**Scheme 4:**
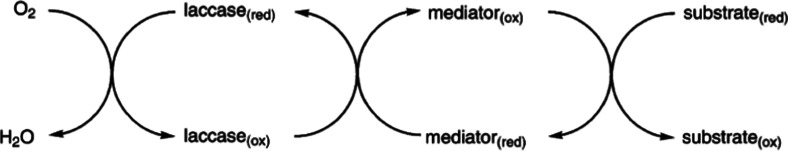
Laccase-catalyzed redox mechanism for substrate oxidation in the presence of chemical mediators.

When a direct oxidation is not achievable due to redox potential incompatibility or steric hindrance of the substrate, chemical mediators can be employed with success. In fact, these molecules are easily oxidized by laccases producing radicals with high *E*^0^; these can diffuse away from the enzyme active site thus allowing oxidation of substrates with limited access to the active site or with high redox potential such as aliphatic, benzylic and allylic alcohols (Scheme 4).^81,82^

Compounds such as (ABTS), 1-hydroxybenzotriazole (HBT) and (2,2,6,6-tetramethylpiperidin-1-yl)oxyl radical (TEMPO) are the most frequently employed mediators, resulting able to reduce reaction time and to improve the yield as well. The laccase–TEMPO system can selectively oxidize the primary hydroxy group over secondary alcohol functions of mono- and oligosaccharides as well as bioactive glycosides under mild conditions.^76^ Thus, the use of mediators in laccase-mediated applications has considerably increased the number of oxidizable substrates and the type of organic derivatives that can be obtained.^83^
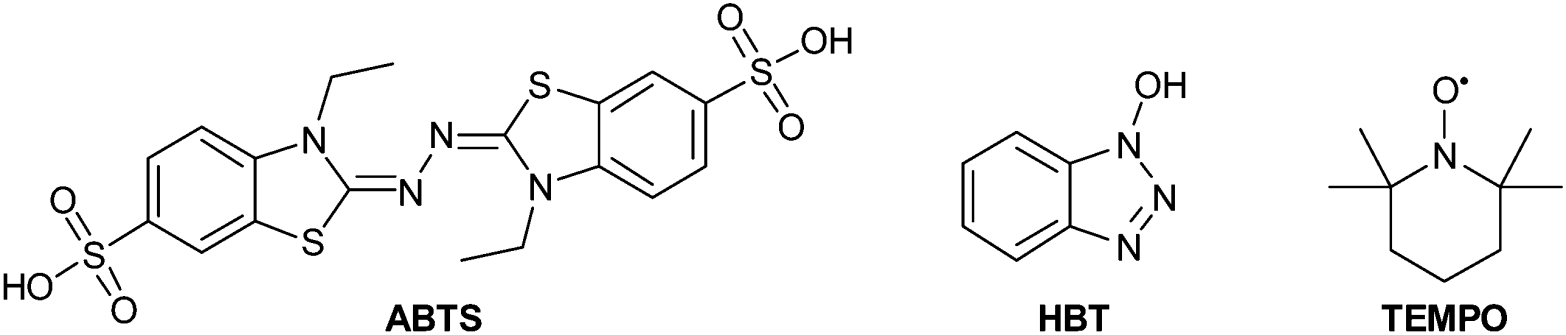


**Fig. 4:**
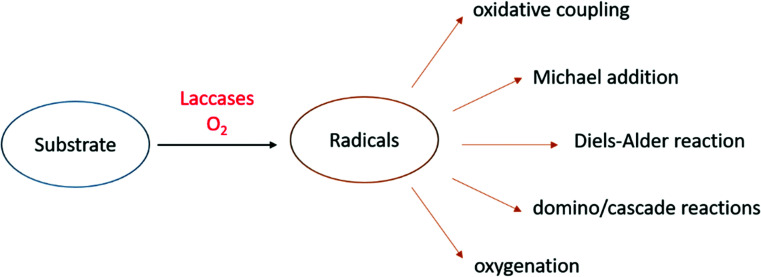
Selected chemical transformations of laccase-formed organic radicals.

To date, laccases have been exploited to several chemical transformations (Fig. 4),^84^ including the oxidative coupling of a large variety of phenolic substrates to yield dimeric compounds. In addition to these “conventional substrates” other compounds such as steroid hormones, alkaloids, antibiotics, and others have been employed in laccase-mediated reactions.^85^

An alternative approach in the use of laccase in organic synthesis is the oxidation of phenolic substrates to the respective quinone structures. The quinonoid derivative is then reacted with other molecules to provide a plethora of organic compounds of pharmaceutical interest. Precisely oxidative cyclocondensation catalysed by laccase can afford several nitrogen-based heterocyclic products:^86^ benzimidazoles, benzothiazoles, quinazolinones, phenazines, and phenoxazinones with biological activity. Michael addiction of primary amines to quinoid intermediate can yield bioactive aminoquinones or novel penicillins.^87^ In a similar manner, benzofuran derivatives can be obtained from catechols and 1,3-dicarbonyl compounds *via* a laccase-mediated oxidation/Michael addition cascade reaction.^88^ Naphthoquinones can be obtained *via* the Diels–Alder reaction of dienes with quinones generated *in situ* by laccase. Finally, with non-phenolic compounds, laccase-mediator systems (LMS) are involved in the oxidation of activated alcohols to ketones or aldehydes.^89^

Moreover, several efforts have been made to determine the ability of laccases from fungi, bacteria and plants towards specific substrates and reactions. Detailed comparisons among enzymes from different sources, in addition to the evaluation of different type of laccases from the same origin, have been reported and commented in well-organized papers.^12,14,76,90^

## 3. Lignans and related compounds

Lignans, neolignans and related compounds are a family of natural products widely distributed in higher plants and characterized by a noticeable structural diversity, with a comparable variety of biological properties.^47^ These plant secondary metabolites have been properly indicated as “a reservoir of biologically active substances”, given their promising properties, including antitumor, antimicrobial, antiviral, anti-inflammatory, neuroprotective, estrogenic and antioxidant activity.^47^

The interest in lignans and neolignans has led to the synthesis of hundreds of analogues inspired by natural leads, and the increasing attention for eco-friendly synthetic methods prompted several research groups to employ laccase-mediated synthesis. In the following sections, we report some selected examples of these syntheses.

### 3.1 Lignans

**Scheme 5:**
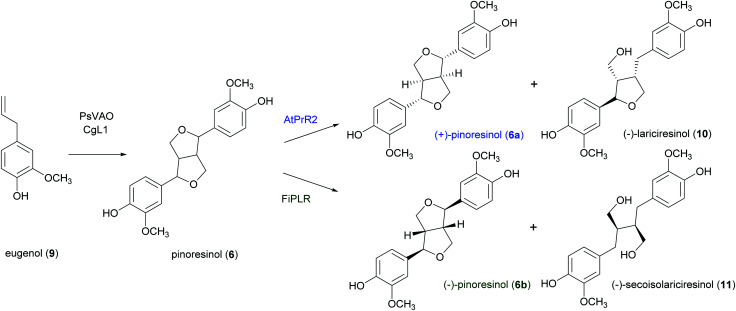
*In vivo* one-pot “one-cell” cascade reaction for the enantiospecific synthesis of enantiopure (+)- and (−)-pinoresinol.

A well-known lignan found in sesame seeds and extra-virgin olive oil is pinoresinol (6). It is considered a high-value product useful for preventing or treating cancer, hyperglycemia, skin pigmentation, microvascular damage, fungal infections and AIDS:^91^6 is also reported as a phytoestrogenic, antioxidant and anti-inflammatory agent.^92^ Enantiopure (+)-pinoresinol (6a) can be isolated from dried ground perisperms of *Sesamum indicum* in low quantities (400 mg from 15 kg; 0.003% yield) with an extraction procedure conducted at room temperature with solvents of increasing polarity.^93^ (−)-Pinoresinol (6b) can be obtained from stems and leaves of *Daphne odora and D. genkwa* after hot MeOH extraction followed by incubation with β-glucosidase in 0.1 M acetate buffer (pH 5.0) at 37 °C for 24 h and subsequent extraction with CH_2_C1_2_. The extraction procedure allowed to obtain an extraction yield of 0.016% calculated over fresh weight and an e.e. of 88%.^93,94^ The marked biological properties and low extraction yields stimulated the synthesis of 6. However, in laboratory syntheses, the absence of a DIR leads to a racemic mixture (Scheme 3). A recent paper reports a one-pot synthesis of racemic pinoresinol from eugenol through an enzymatic cascade combining the *Penicillium simplicissimum* vanillyl-alcohol oxidase (PsVAO) with the *Corynebacterium glutamicum* laccase (CgL1).^95^ PsVAO converts eugenol (9) into coniferyl alcohol (5), the biosynthetic precursor of 6. CgL1 catalyses the 8–8′ coupling of two coniferyl radicals to give the cyclized product 6. The reaction parameters, such as enzyme, reaction time, pH value, enzyme/substrate ratio, and additives were optimized for a semi-preparative scale-up as followings: mM of eugenol (9), 20% (v/v) TBME, and PsVAO/CgL1 in a ratio of 1 : 5. After 120 h a 47% conversion of eugenol was achieved and lignan 6 was obtained with 13%yield.^95^ The same research group obtained more recently enantiopure 6a and 6b with 12 and 6.1% yield respectively. by employing an *in vivo* one-pot “two-cell” sequential cascade reaction. Namely, in a first step an *Escherichia coli* whole-cell biocatalytic system harbouring PsVAO and CgL1, was employed with 10 mM eugenol (9). This procedure yielded 100% conversion of eugenol and was followed by a second step with *E. coli* cells harbouring an enantiospecific pinoresinol reductase from *Arabidopsis thaliana* (AtPrR2) able to convert 6b into (−)-lariciresinol (10), thus affording 6a with 98% e.e. (Scheme 5).^91^ In a similar procedure, the enantiospecific reductase from *Forsythia intermedia* (FiPLR) allowed to obtain enantiopure 6b with 97% e.e. Of note, from this reaction (−)-secoisolariciresinol (11), another bioactive lignan,^96^ was obtained as side product with 37.7% yield and 99% e.e.^91^

**Scheme 6:**
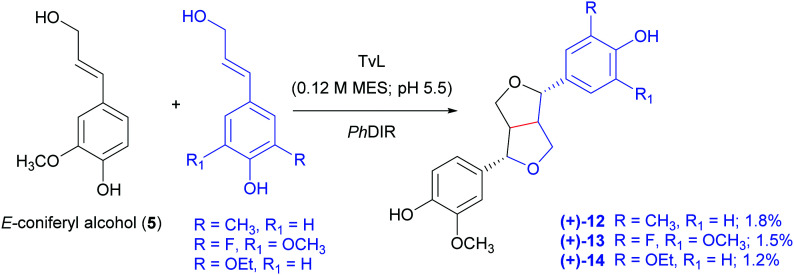
TvL- and *Ph*DIR-mediated heterocoupling of coniferyl alcohol with selected synthetic analogues. MES: 2-(*N*-morpholino)ethanesulfonic acid. The monomers are solubilized in DMSO.

In a very recent paper, a combination of TvL and a DIR from *Podophyllum hexandrum* (*Ph*DIR) was employed to achieve the regio- and enantioselective heterocoupling of natural and non-natural monolignols (Scheme 6).^97^ The aim of the work was to obtain analogues of 6a as possible precursors of anticancer agents inspired by etoposide but bearing different substituents in the aromatic ring: this could prevent demethylation by the human liver enzyme CYP3A4, that causes the formation of a quinone with altered reactivity. By carefully optimising TvL-*Ph*DIR preparatory-scale reactions with 5 and its synthetic analogues shaken at 300 rpm at 30 °C, the authors obtained three non-natural (+)-pinoresinol analogues (12–14) with 1.8, 1.5 and 1.2% yield respectively, and an e.e. ranging from 73 to 93%.

The majority of laccase-mediated syntheses of lignans and related compounds have been carried out with fungal laccases; however, bacterial laccases are promising as industrial biocatalysts due to the ease of expression in host such as *Escherichia coli*. In this context, a two-domain bacterial laccase from *Streptomyces coelicolor* A3 (0.64 U mL^−1^) was effectively employed to catalyse the oxidative dimerization of caffeic acid (10 mM) in 50 mM sodium phosphate buffer (pH 7.5) at 25 °C.^98^ This reaction afforded an unsymmetrical 8–8′ caffeic acid dimer, previously known as phellinsin A (15), a natural lignan found in culture broth of *Phellinus* sp. and reported as antioxidant.^99^ Although different solvents were evaluated, 80% ethyl acetate allowed to obtain a cleaner reaction mixture, thus making it easier for purification. Compared to caffeic acid, phellinsin A showed significantly improved antioxidant activity, in both DPPH and TEAC assays, better solubility in aqueous media and remarkable stability in acid environments.

Benzoxanthenones are a rare group of lignans with an oxygenated tetracyclic skeleton. Carpanone (16), isolated from the bark of carpano tree (*Cinnamomum* sp.) and sauchinone (17) are two representative members. The lignan 17 has been reported as an hepatoprotective, antihypertensive, immunosuppressive and anti-inflammatory agent.^100^ Some synthetic analogues of 16 were able to perturb the secretory pathway by inhibiting exocytosis from the Golgi apparatus.^101^
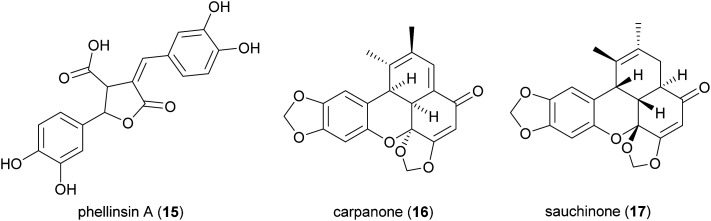


**Scheme 7:**
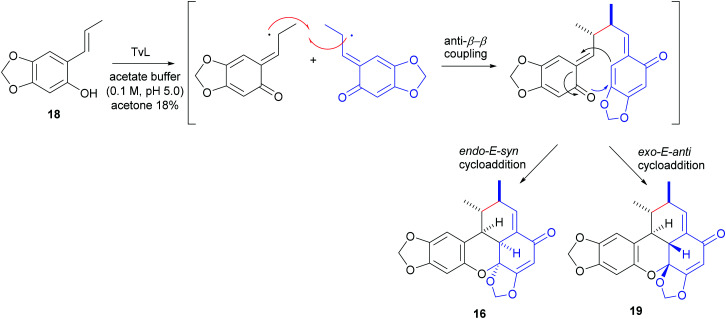
TvL-mediated synthesis of carpanone and it diastereoisomer 19.

An elegant, biomimetic synthesis of carpanone has been carried out starting from (*E*)-2-propenylsesamol (18), whose oxidative dimerization catalysed by TvL in 18% acetone afforded carpanone (16) and its diastereoisomer 19 with a global yield of 68% in a 9 : 1 ratio (Scheme 7).^100^

The formation of benzoxanthenone structures can be explained through a domino phenol oxidation/anti-8,8′-radical coupling/intramolecular hetero-Diels–Alder reaction. In the formation of carpanone, cycloaddition proceeds *via* the *endo-E-syn* transition state which is more energetically favoured due to the less steric strain respect to the *exo-E-anti* transition state which afford compound 19, thus justifying the prevalent formation of carpanone.

### 3.2 Neolignans

2,3-Dihydrobenzofurans, a major group within neolignans, have been widely reported in the literature for their interesting biological activity, including cytotoxic, anti-inflammatory, antioxidant and α-glucosidase inhibitory activity.^47^ The biomimetic syntheses of 2,3-dihydrobenzofurans causes the formation of two novel stereogenic centres and the coupling reaction occurs with diastereoselectivity (the less hindered *trans*-substituted cycle is preferred), but without enantioselectivity, leading to racemic mixtures (see Scheme 3, Section 2). Ferulic acid dimerization catalysed by *Trametes pubescens* (TpL) laccase in a monophasic system afforded as major product with 11.2% yield the 2,3-dihydrobenzofuran 20, a more effective antioxidant than the monomer.^65^ In a subsequent work, 20 showed antioxidant and antiangiogenic activity, and affected granulosa cell viability redox status and steroidogenesis.^102^ Oxidative coupling of some ferulic esters in the presence of TvL or *Agaricus bisporus* laccase (4 U mL^−1^) in acetate buffer (0.1 M, pH 5.0), afforded dihydrobenzofurans (21–24) with 23–25% yields.^103^ Other 8-5′-dimers (25–27) obtained from caffeic acid esters revealed to be microsomal prostaglandin E2 synthase (mPGES-1) inhibitors in the low-micromolar range, being privileged structures for the development of new selective anti-inflammatory agents.^104^ In a chemoenzymatic synthesis of glycosylated 2,3-dihydrobenzofurans, the key step was a dimerization of phenylpropanoid glucosides (0.05 M) in acetate buffer (0.02 M, pH 5.0) mediated by 4.6 U m L^−1^ TvL, which afforded novel water soluble dihydrobenzofurans with 35% yield.^19^ The substitution of d-glucose (28) with l-glucose (29) or rutinose (30) minimally affected the stereochemistry of the reaction, and the best d.e. obtained was 21%.
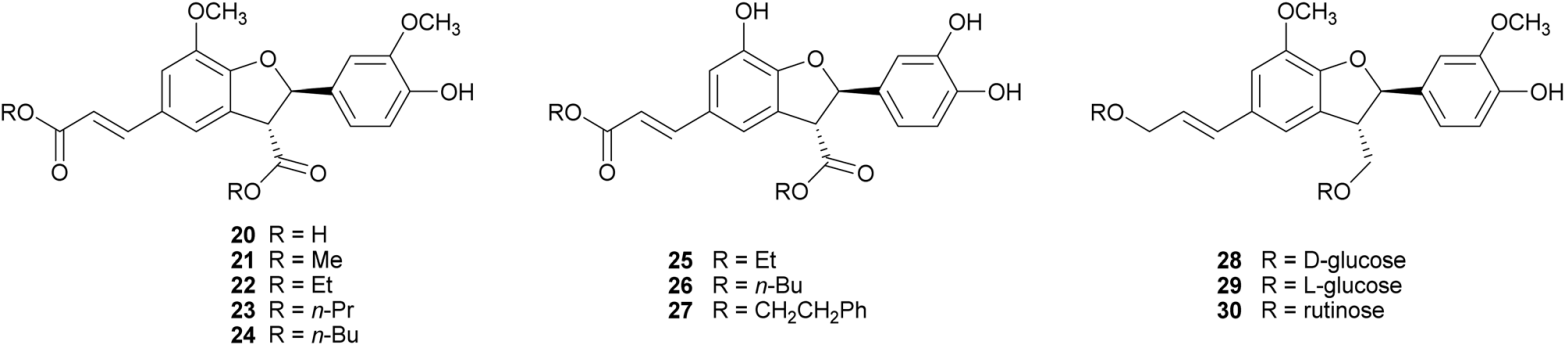


**Scheme 8:**
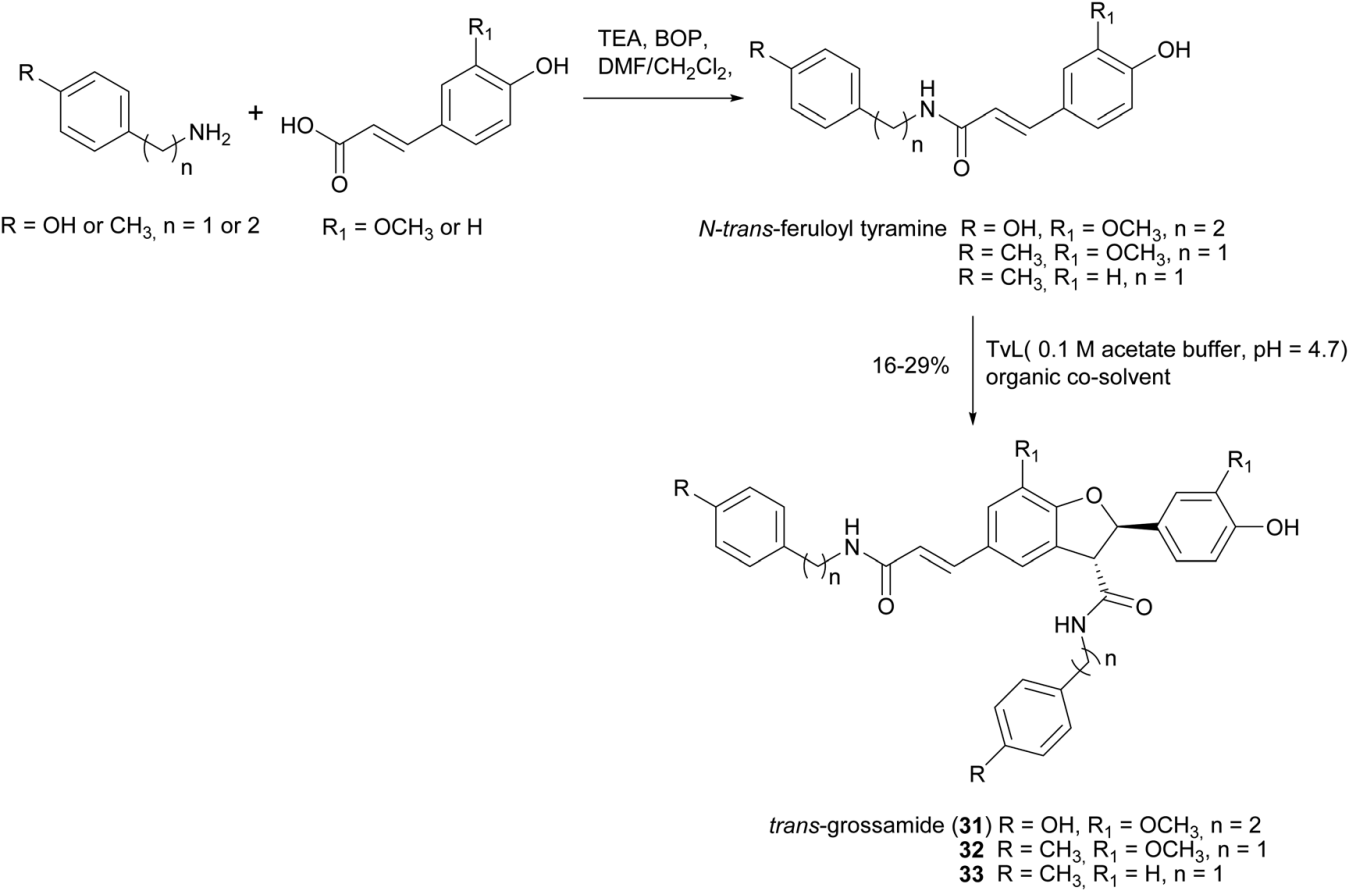
Chemo-enzymatic synthesis of neolignanamides 31–33. TEA: triethyl amine; BOP: benzotriazole-1-yl-oxy-tris-(dimethylamino)-phosphonium hexafluorophosphate. See Scheme 1b for the mechanism of dihybrobenzofuran-type neolignan formation.

Lignanamides and neolignanamides constitute subclasses of lignans incorporating an amide group in their structure; these compounds display promising biological properties, including anti-inflammatory, antioxidant, antibacterial, antitumor, neuroprotective, anti-melanogenic, anti-hyperlipidaemic and insect antifeedant activity.^105^ A biomimetic oxidative coupling of the natural *N-trans*-feruloyl tyramine in the presence of TvL (Scheme 8) afforded with 16% yield *trans*-grossamide (31), a natural neolignanamide with a dihydrobenzofuran core, found in *Hyosciamum niger* and other plants, and reported as anti-inflammatory, mild cytotoxic, antifungal and adrenergic receptor antagonist.^106^ In the same work, further bioinspired amides of the common cinnamic acids were synthetized and submitted to biomimetic dimerization, thus affording eight previously unreported neolignanamides. The enzymatic dimerization was achieved with TvL (in acetate buffer, 0.1 M, pH = 4.7) and ethyl acetate and DMSO were employed as co-solvents. In that conditions, neolignans such as 32 and 33 were obtained with 16% and 29% yield respectively (Scheme 8). The racemates were evaluated as antiproliferative agents against Caco-2 (colon carcinoma), MCF-7 (mammary carcinoma), and PC-3 (prostate cancer) human cells. Interestingly, *trans*-grossamide was only moderately active, whereas its strictly related analogues 32 and 33, the most lipophilic in the series, were potently active, with GI_50_ values comparable or lower than that of the anticancer drug 5-fluorouracil. A study of the effect on the cell cycle revealed that 32 was an inducer of cancer cell cycle arrest and apoptosis.

## 4. Dimeric stilbenoids

Stilbenoids, whose most representative member is resveratrol (34), are generated by a biosynthetic pathway distinct from those of other polyphenols, such as phenylpropanoids or flavonoids. Resveratrol has reached an impressive level of popularity mainly due to a variety of beneficial biological properties, including anti-inflammatory action as well as prevention of cardiovascular diseases and colon cancer.^107,108^ Further stilbenoids, including isorhapontigenin (35), have been reported for promising pharmacological properties,^109^ and this attracted the attention of researchers also to the dimeric and oligomeric stilbenoids, narrowly distributed in the Plant Kingdom, and only lately recognized as natural products with significant biological properties, including antioxidant, antimicrobial, anti-inflammatory and antitumor activity.^110,111^ It is generally accepted that the biosynthesis of dimeric stilbenoids proceeds through phenolic oxidative coupling, although different mechanisms have been proposed. Among the resveratrol dehydrodimers found in grape, δ-viniferin (36) was found moderately cytotoxic against CEM (human lymphoblastoid) cells,^112^ and ε-viniferin (37) has been evaluated as anticancer, anti-diarrheic and anti-Alzheimer agent.^113^ Very recently, 36 and its methoxylated analogue 38 (pterostilbene dehydrodimer) proved to be DNA-binding agents more effective than their monomeric parent compounds, also showing cytotoxic activity in the micromolar range on several cancer cell lines.^114^ Dimer 37 has been reported to reduce hyperglycemia and hyperlipidemia through activation of AMPK *in vivo*.^113^
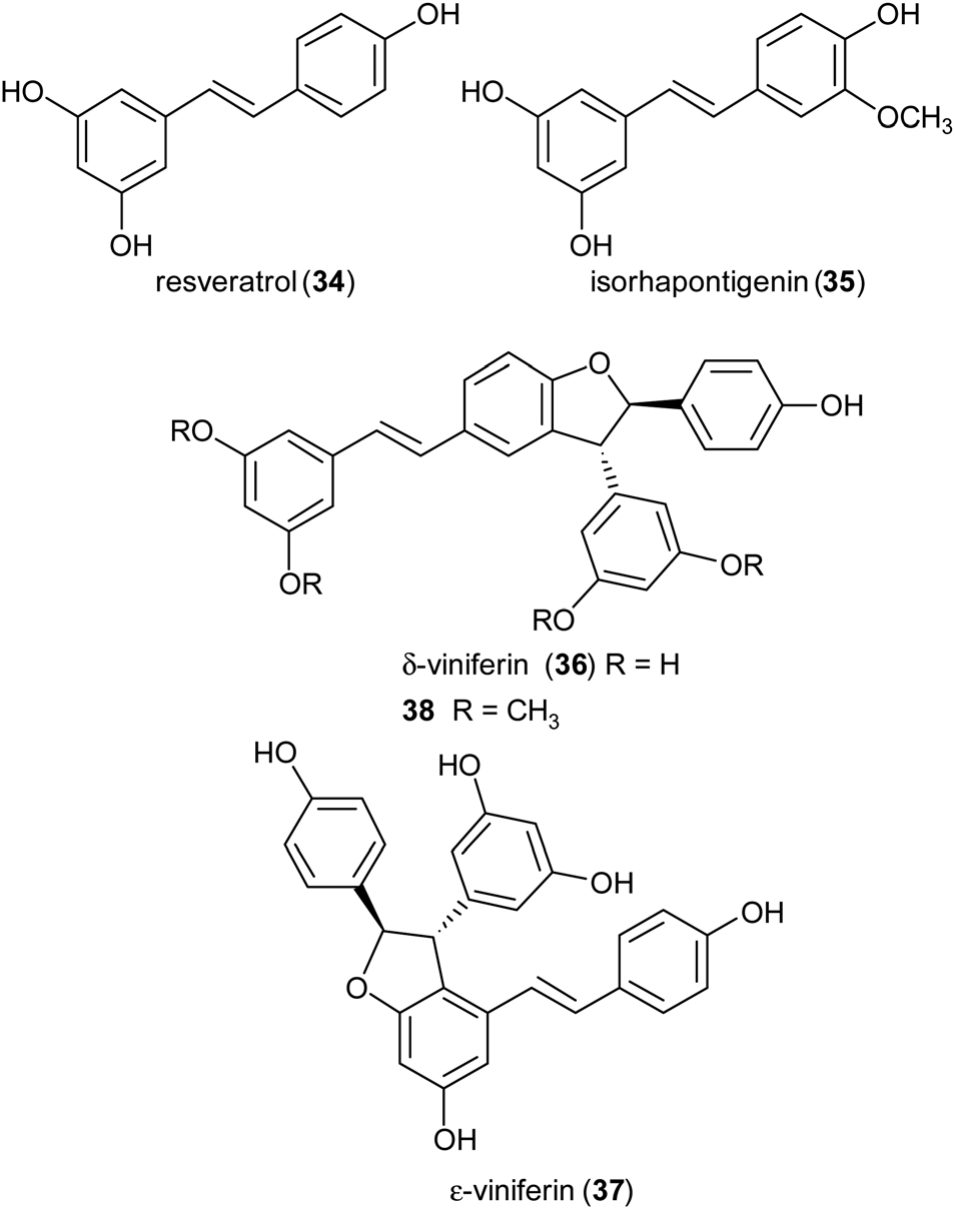


**Scheme 9:**
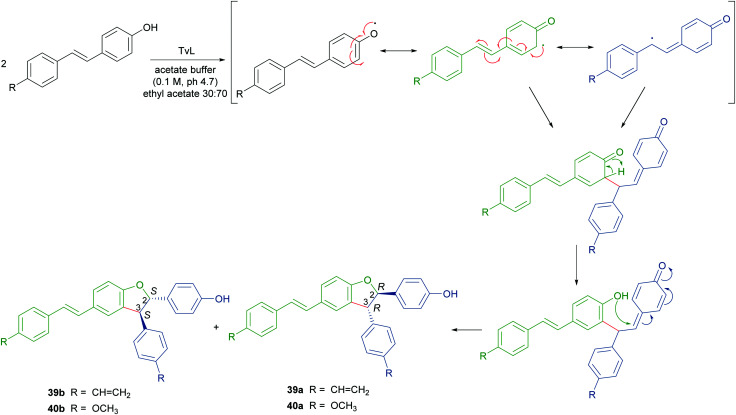
Biomimetic synthesis of dimeric stilbenoids mediated by TvL and proposed mechanism of formation.

To obtain both enantiomers of 36, the 3-*O*-glucoside of resveratrol (piceid) was employed as starting material.^66^ As in the syntheses of dihydrobenzofuran neolignans, also the laccase-mediated oxidative coupling of stilbenoids occurs with diastereoselectivity but lacks of enantioselectivity, so racemic mixtures of the *trans*-disubstituted dimers are formed. Dimerization of piceid in the presence of TvL (in 20 mM acetate buffer, pH 5.0) at 25 °C in an open flask for 210 min, afforded the corresponding (±)-*trans*-dehydrodimer with 45.7% yield. The diastereomeric mixture was submitted to chiral resolution on HPLC. Finally, starting from the pure diastereomers the cellulase from *Trichoderma viride* afforded the 2*R*,3*R*-enantiomer 36a and 2*S*,3*S*-enantiomer 36b with e.e. > 99% e.e. 98.3% respectively. Starting from synthetic stilbenoids some of us carried out a biomimetic synthesis of seven resveratrol-related dehydrodimers inspired by δ-viniferin. The reaction, performed with TvL (10 U mL^−1^) in a biphasic system of acetate buffer (pH 4.7) and ethyl acetate, afforded 39 and 40 with 24 and 65% yield respectively.^115^ In Scheme 9 the proposed mechanism of formation of the two representative dimers 39 and 40 is reported. The products were evaluated as antiproliferative agents against SW480 human colon cancer cells; interestingly, the only two inactive dimers (IC_50_ > 100 μM) were those bearing a methoxy group in the position *ortho* to the C-4 hydroxy group; the racemates 39 and 40 showed respectively IC_50_ values of 22.3 and 33.4 μM. Chiral HPLC resolved the racemates, and the absolute configurations at C-2 and C-3 of dihydrobenzofuran core of the pure enantiomers were determined through analysis of their CD spectra. In the bioassay on SW480 cells, the most potent enantiomers were those with 7*R*,8*R* absolute configurations (39a: IC_50_ = 20.0 μM; 40a: IC_50_ = 19.9 μM), but a defined configuration of the stereogenic centres does not appear to be an essential structural requirement for the activity.

A group of eleven *trans*-dihydrobenzofurans, including CN, NO_2_, SCH_3_ and OCH_3_ as aryl substituents, was obtained through TvL-mediated oxidation of chemically synthesized (*E*)-4-styrylphenols. The reaction was carried with TvL (42 U mmol^−1^ substrate), in acetate buffer (50 mM, pH 4.5) and acetone (1 : 1) at 27 °C, shaking at 160 rpm for 24 h, and afforded the *trans*-dihydrobenzofurans with 33–74% yields.^116^ In addition, reduction of the NO_2_ substituent or introduction of the *N*,*N*-dimethylethanamine pendant onto phenolic OH afforded other amine-substituted dimers such as 41. Because heat shock protein (Hsp90) is a promising target for the treatment of vascular, neurodegenerative and cancer disease, all compounds were tested *in vitro* as potential allosteric modulators of Hsp90 ATPase activity. The most potent racemic dimers were ε-viniferin (37) and dimer 41. *In vitro* results supported by docking studies indicated that the two enantiomers of ε-viniferin have a comparable activity towards Hsp90.
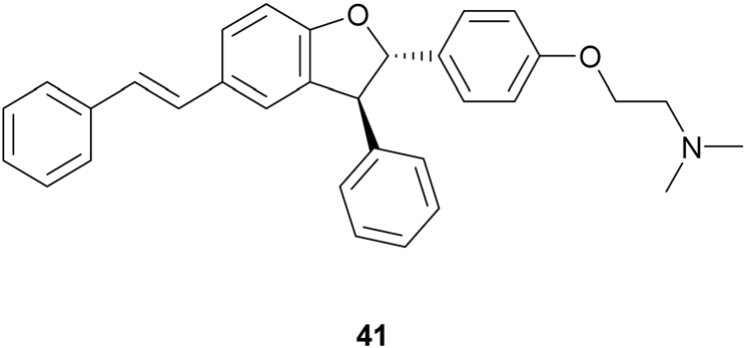


**Scheme 10:**
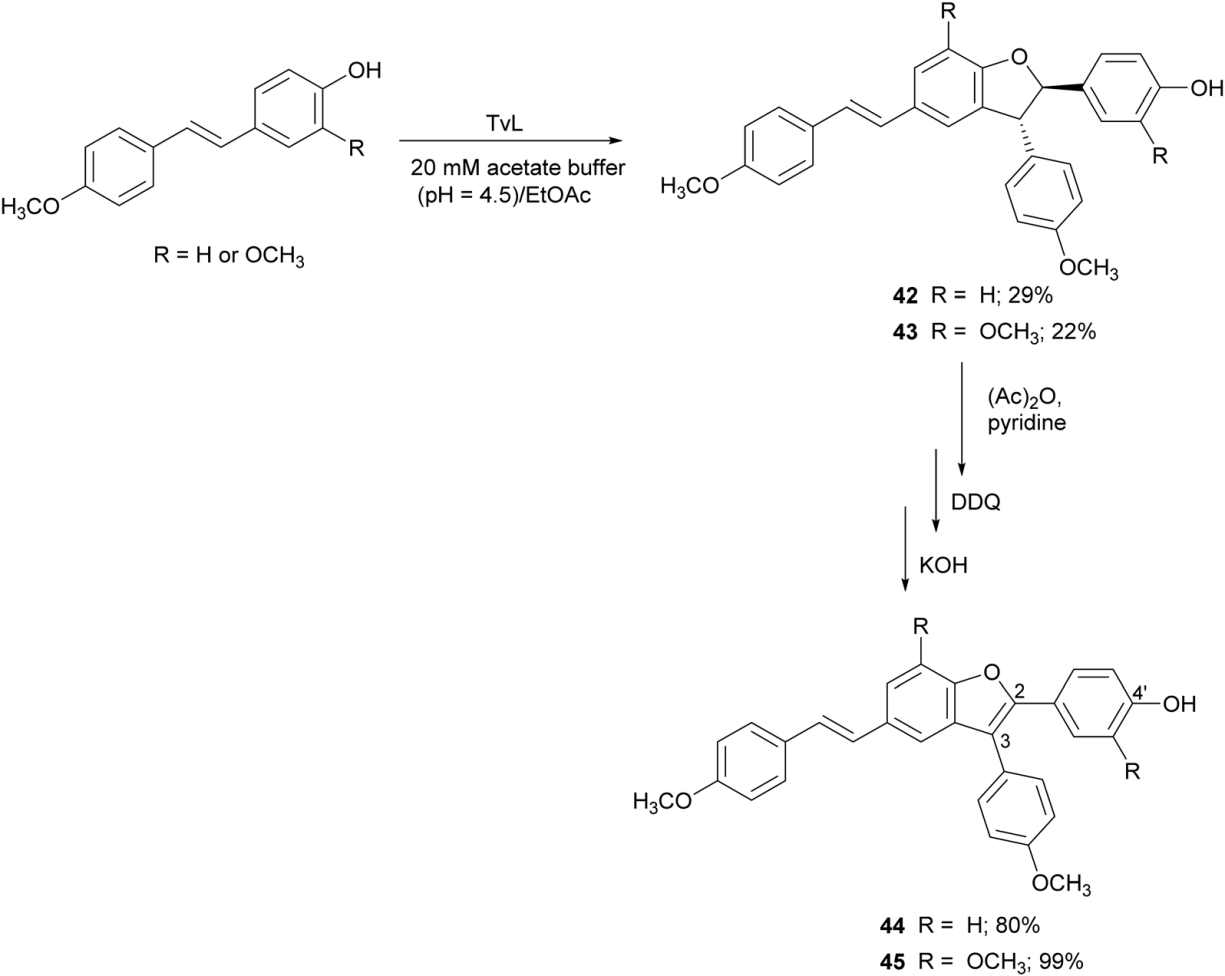
TvL-mediated synthesis of dimeric stilbenoids and their 2,3-diarylbenzo[*b*]furan analogues. DDQ = 2,3-dichloro-5,6-dicyano-*p*-benzoquinone.

Further resveratrol-related dehydrodimers including compounds 42 and 43 were obtained through TvL-mediated oxidative coupling in a biphasic system with the substrate dissolved in ethyl acetate and the laccase (40 U) in 20 mM acetate buffer (pH 4.5). The products 42 and 43 were obtained with 29 and 22% yield, and were subsequently oxidized with 2,3-dichloro-5,6-dicyano-*p*-benzoquinone (DDQ) to 2,3-diarylbenzo[*b*]furan analogues 44 and 45, employing protection/deprotection steps (Scheme 10).^117^ Both natural and synthetic benzo[*b*]furans are reported in the literature for a variety of biological activities, and some commercial drugs include a benzo[*b*]furan core.^116^ Benzo[*b*]furans 44 and 45 were found to be DPPH radical scavengers more effective than the corresponding dehydrodimers.^117^ This evidence was explained by analysing the structure of the benzofurans. The optimized structure obtained with Polak–Ribière conjugate gradient algorithm pointed out an almost planar structure in 44 and 45 due to the presence of double bond at C2–C3 which leads to a more extended delocalization for the 4′-phenoxy radical to the adjacent ring.^117^

**Scheme 11:**
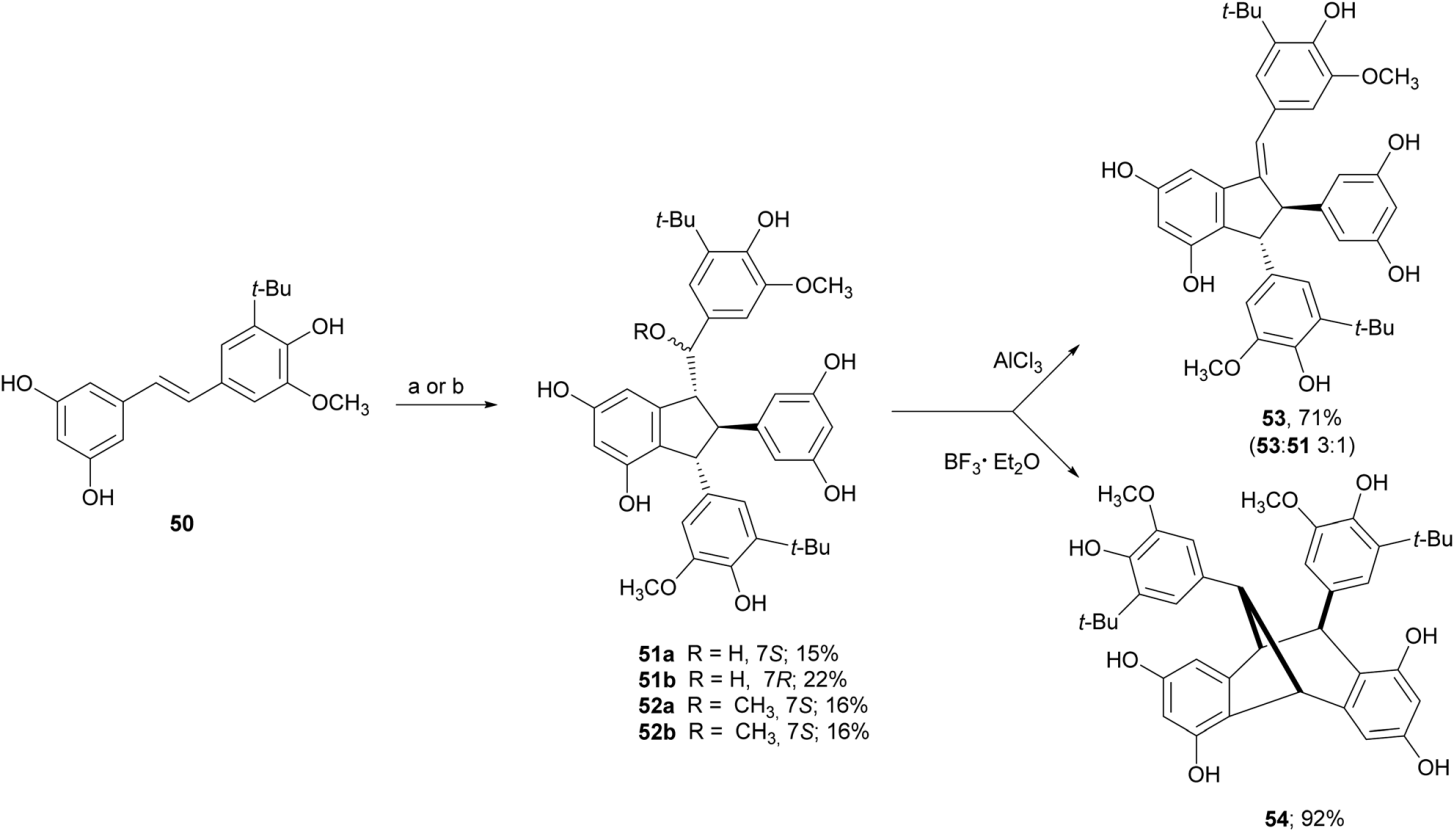
Synthesis of isorhapontigenin-based dimers. (a) TvL in acetone : water 2 : 1; (b) HRP in methanol : water (4 : 1).

Due to the lack of regioselectivity in biomimetic oxidative coupling of stilbenoids, minor attention has been devoted to the synthesis of dimeric scaffolds different from those reported above, notwithstanding the variety of bioactive natural stilbenoids, exemplified by quadrangularin A (46), gnetulin (47), gnetuhainin I (48), and gnemonol M (49). However, employing an isorhapontigenin-based stilbenoid bearing a bulky *t*-butyl group (50), the diastereoisomers 51 and 52, butylated analogues of gnetuhainin I, were obtained with 15–22% yield employing TvL or horseradish peroxidase (HRP) (Scheme 11).^118^ Interestingly, by treating 51 or 52 with Lewis acids such as AlCl_3_ or BF_3_·Et_2_O, other isorhaponthigenin-based dimers (53 and 54) were obtained as unnatural analogues of 46 and 49. In particular, the dimer 53 was obtained with 71% yield as inseparable mixture with the dimer 51 (ratio 3 : 1) employing 0.5 equivalents of AlCl_3_, whereas, compound 54 was isolated with 92% yield employing 4 equivalents of BF3·Et_2_O.
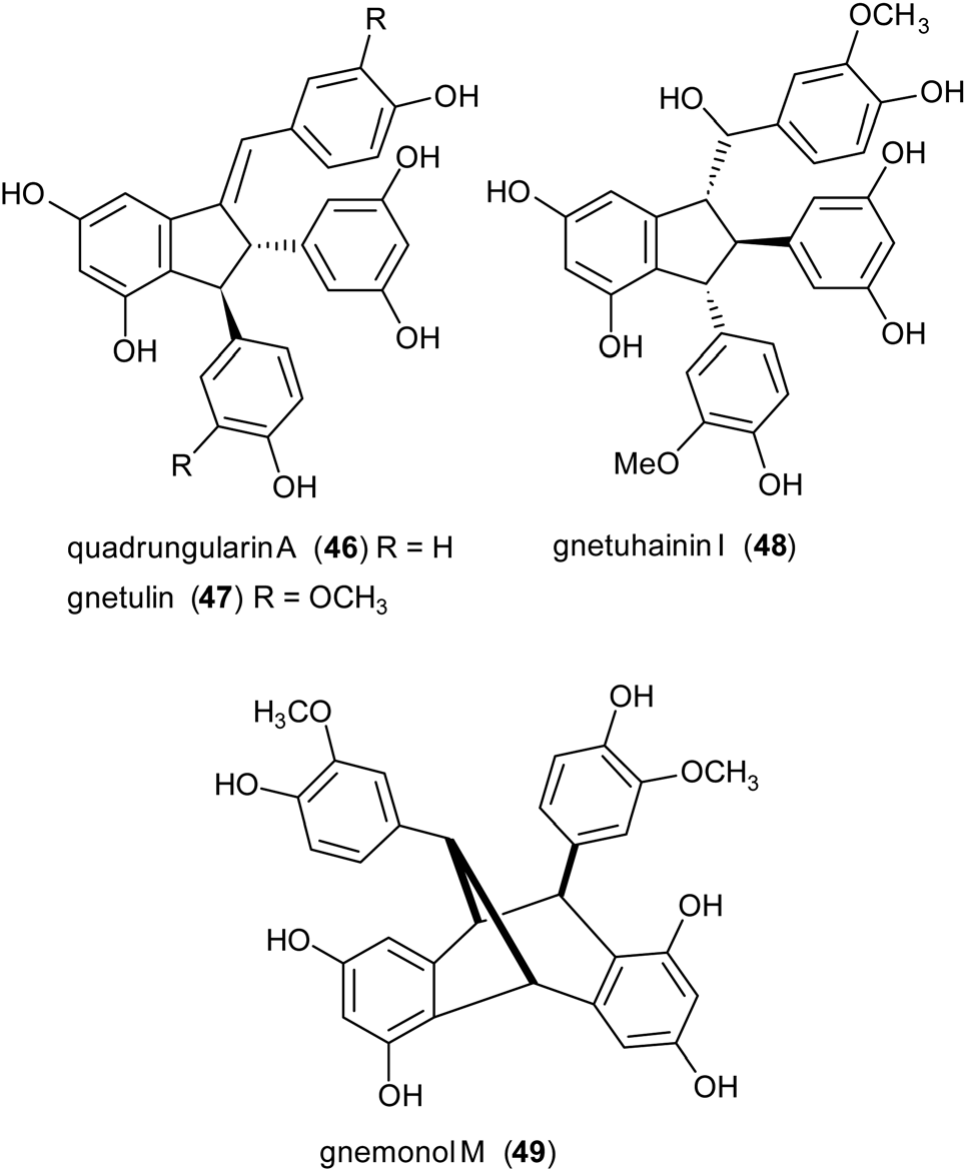


**Scheme 12:**
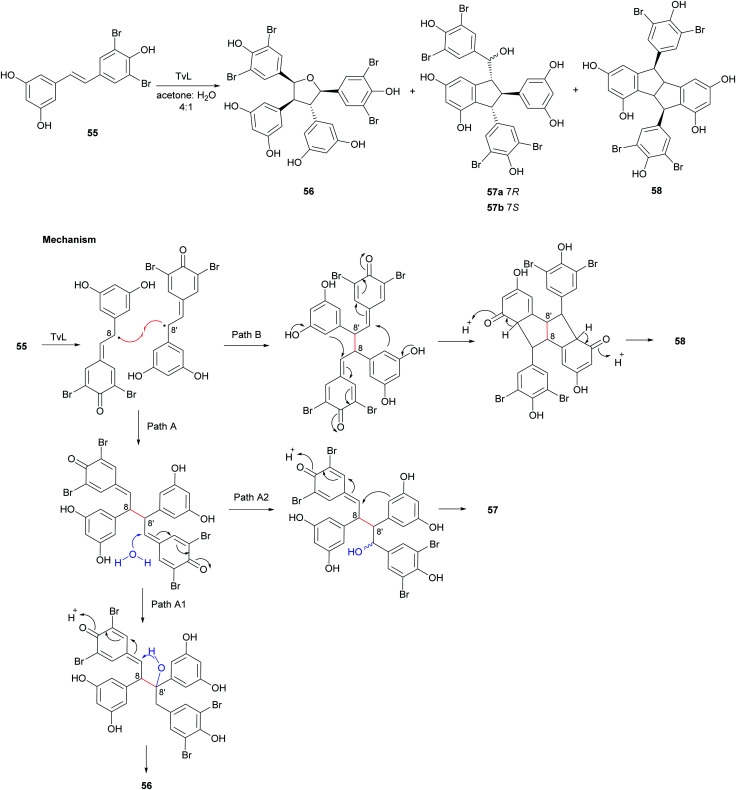
Coupling products from the TvL-mediated oxidation of 55 and proposed mechanism of formation of products 56–58.

In a later work,^119^ regioselective oxidative coupling of 3′,5′-dibromoresveratrol (55) was performed employing HRP or TvL. In particular, the reaction performed in TvL and in acetone:water afforded the mixture of dimers 56–58; the proposed mechanism of formation proceeds *via* 8–8′-coupling and subsequent rearrangement of a quinone methide intermediate in different ways (Scheme 12). The reaction mixture was purified on a silica gel column chromatography (CH_2_Cl_2_ : MeOH; 25 : 1) to give a mixture of 56, 57a and 57b (1 : 3 : 1, 45% global yield) and the dimer 58 (15% yield).

## 5. Dimeric flavonoids

**Scheme 13:**
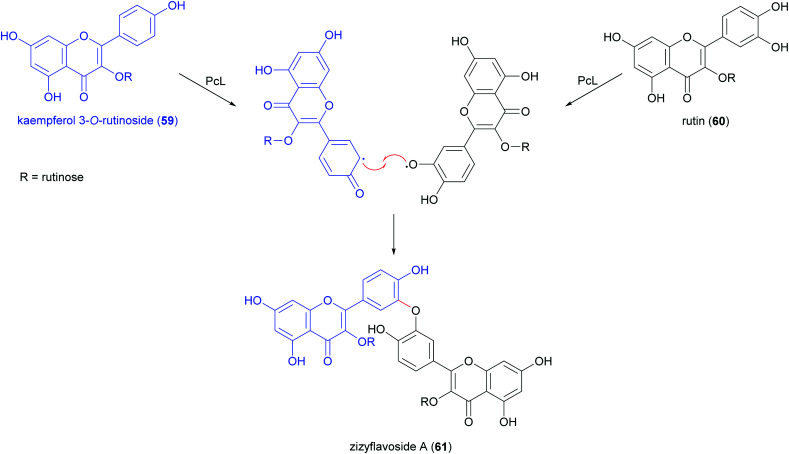
Biomimetic synthesis of natural zizyflavoside A and proposed mechanism of formation.

Biflavonoids are a subclass of the plant flavonoid family. Notwithstanding the limited number of natural biflavonoids reported in the literature, they have been investigated for a variety of pharmacological properties: these dimeric compounds are endowed with anti-inflammatory, antioxidant and antiprotozoal activity;^120^ in addition to other beneficial properties, they have a role in the management of obesity, diabetes, cognitive disorders and cancer.^121^ Among the few papers dealing with laccase-mediated synthesis of biflavonoids, it is worth of citation here the oxidative heterocoupling of kaempferol-3-*O*-rutinoside (59) and quercetin-3-*O*-rutinoside (rutin, 60) mediated by laccase from *Pycnoporus cinnabarinus* (PcL), which afforded the natural biflavonoid zizyflavoside A (61) with 8% yield (Scheme 13) much better than the very low yield (0.0012%) obtained by extraction from *Zizyphus spina-christi* (L.) wild leaves.^122^

Laccases from the fungi *Pycnoporus coccineus* and *P. sanguineus*, were employed to catalyse the synthesis of a number of oligomers of rutin (60) in the aqueous mixture of glycerol/ethanol/buffer.^123^ The oligomer mixtures were analysed by HPLC-DAD/ESI-MS and the products were characterized as rutin dimers and trimers. Dimers with structure 61 and 62 are the main products of the mixture representing respectively 14% and 35% of all the quantified oligorutins. The oligomer mixtures showed antioxidant, anti-inflammatory and superoxide radical scavenging higher than those of rutin, being also able to inhibit human cyclooxygenase (COX-2) and matrix metalloproteinase (MMP-3).
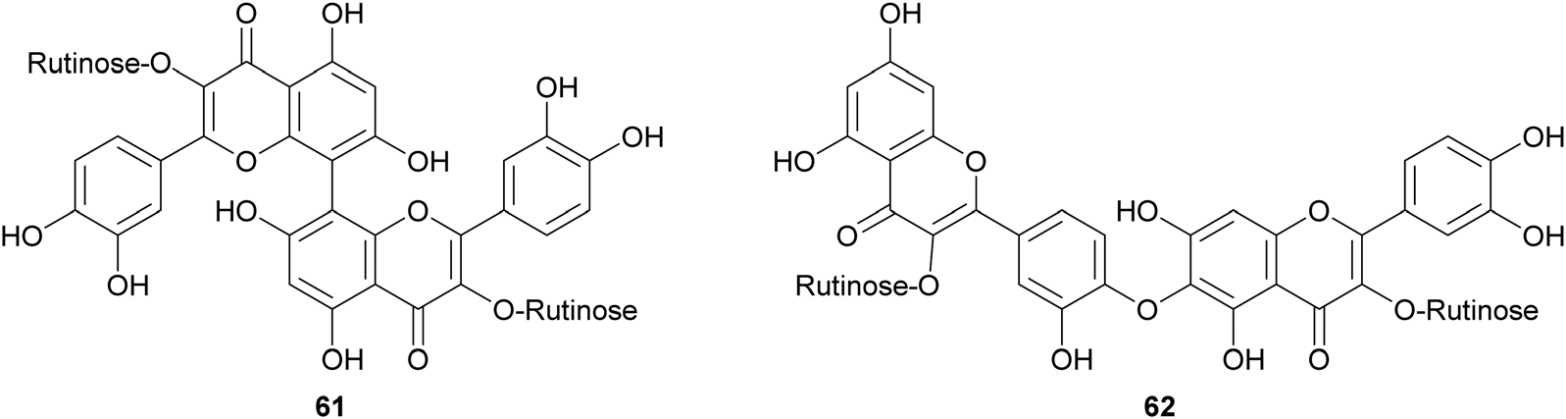


**Scheme 14:**
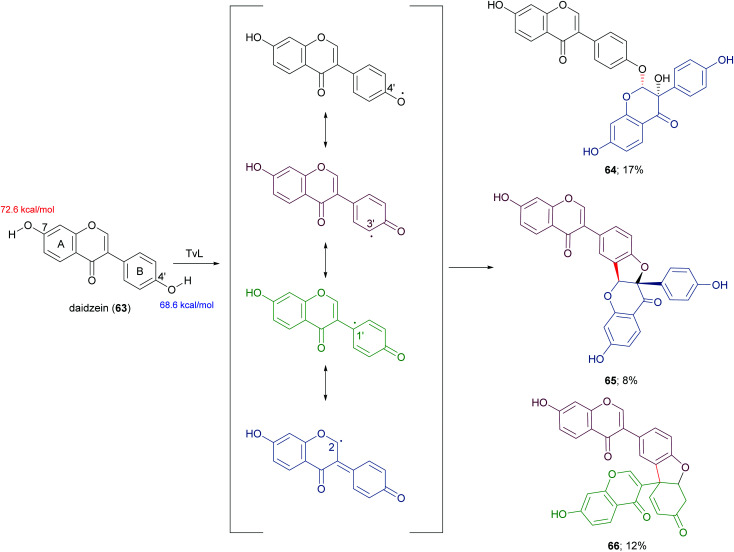
Dimeric products obtained by laccase-mediated coupling of daidzein. Bond energy values (kcal mol^−1^) for the phenolic group at C-7 and C-4′ of 63, are reported in red and blue, respectively.

**Scheme 15:**
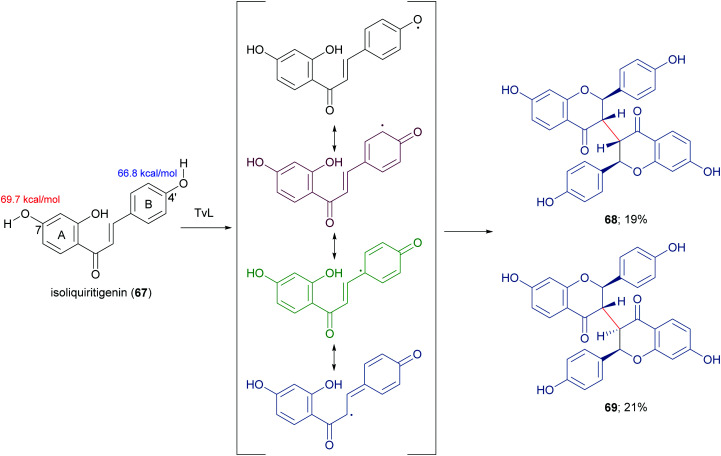
Dimeric flavonoids obtained by laccase-mediated reaction of isoliquiritigenin. Bond energy values (kcal mol^−1^) for the phenolic group at C-7 and C-4′ are reported in red and blue, respectively.

In a more recent work, the biocatalytic oxidation of flavonoids mediated by TvL and HRP was studied.^124^ TvL was dissolved in citric acid–hydrogen phosphate buffer solution (pH 4.0) and incubated at 30 °C with each flavonoid (dissolved in acetone) for 6 h. Different flavonols, isoflavones and chalcones with at least one phenolic group were effective substrates for laccase, however the type of product differed with structure. Namely, kaempferol was converted into a monomeric oxidized product, whereas the isoflavone daidzein (63) afforded dimers 64–66 (Scheme 14, yields in the range 8–17%) and the chalcone isoliquiritigenin (67) gave dimers 68 and 69 (Scheme 15, yields 19–21%). A docking study on laccase showed that most of the flavones studied binds to the surface of laccase, whereas daidzein and isoliquiritigenin bind to the sites close to the T1Cu active centre of the enzyme, justifying the formation of dimers. Of note, OH bond energy analysis revealed the lowest bound energy of 4′-OH in the B-ring of flavones, explaining the predominant formation of dimeric flavonoids onto ring B.

## 6. Biflavonolignans

Flavonolignans are a small group of mixed-biosynthesis lignans, incorporating both flavonoid and phenylpropanoid units. The most frequently reported flavonolignans are constituents of silymarin, an industrial extract of the milk thistle (*Silybum marianum*) seeds. It is a complex of flavonolignans and other polyphenols and is reported as anticancer,^125^ antioxidant, anti-inflammatory, hepatoprotective, neuroprotective and antiviral.^126^ The major components of silymarin include silybin A (70), silybin B (71), silychristin A (72) and silydianin (73). These compounds are antioxidants,^127,128^ have inhibitory properties against the G protein-coupled receptor P2Y12 and block ADP-induced blood platelet activation, thus reducing the risk of heart stroke, vascular diseases and ischemia.^129^
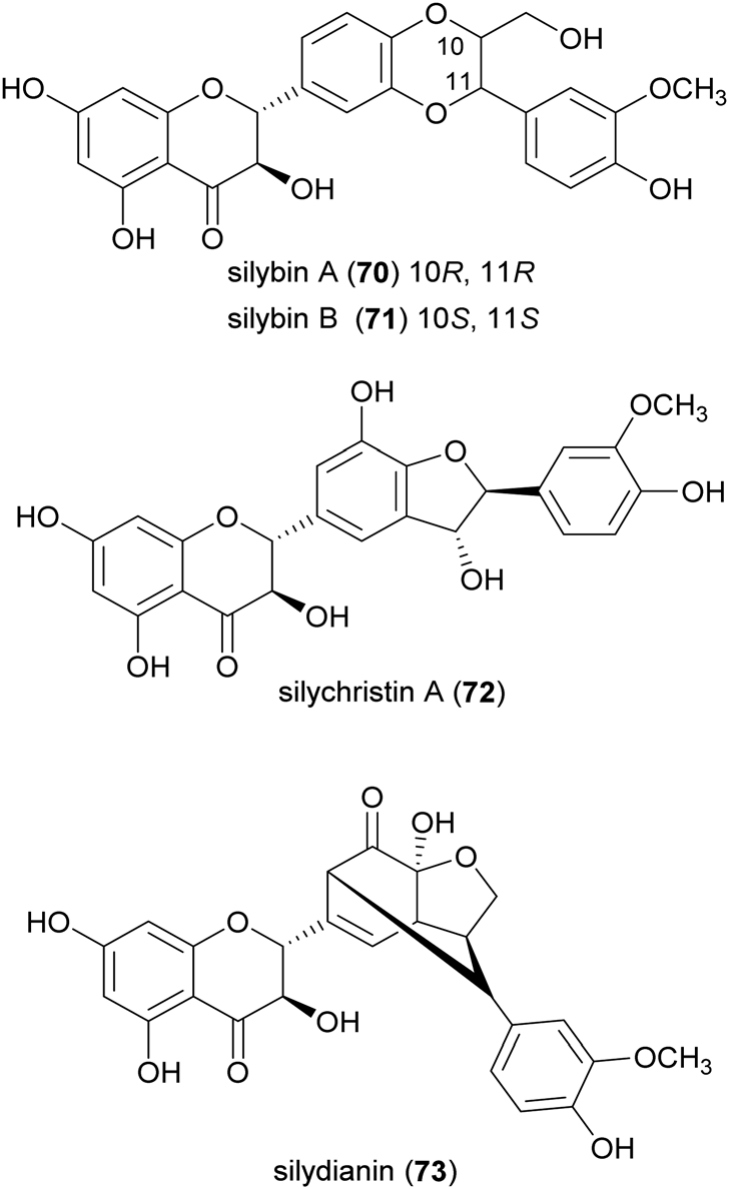


Bi-flavonolignans are an emerging group of synthetic dimers obtained by oxidative coupling of flavonolignans; previous chemo-enzymatic synthesis of silybin dimers afforded products with enhanced antioxidant and antitumor properties.^130^ TvL-mediated dimerization of 70 and 71, dissolved in acetone and DMF and-acetate buffer (20 mM, pH 4.5), afforded a complex mixture of oligomers and polymers. However, after the protection of the substrates as 7-*O*-benzyl derivatives and subsequent deprotection, the C–C homodimers 74 and 75 were obtained with 87.5 and 87.2% yield. Surprisingly, the dimeric flavanolignan of silydianin (76) was obtained with 21.5% yield without further protection step, whereas oxidative coupling of silychristin A (72) gave a complex, inseparable mixture employing both the protected and the unprotected substrate.^127^ The flavonolignans dimers 74–76 were submitted to the DDPH radical scavenging assay and showed an antioxidant activity more than double with respect to that of the parent monomers.
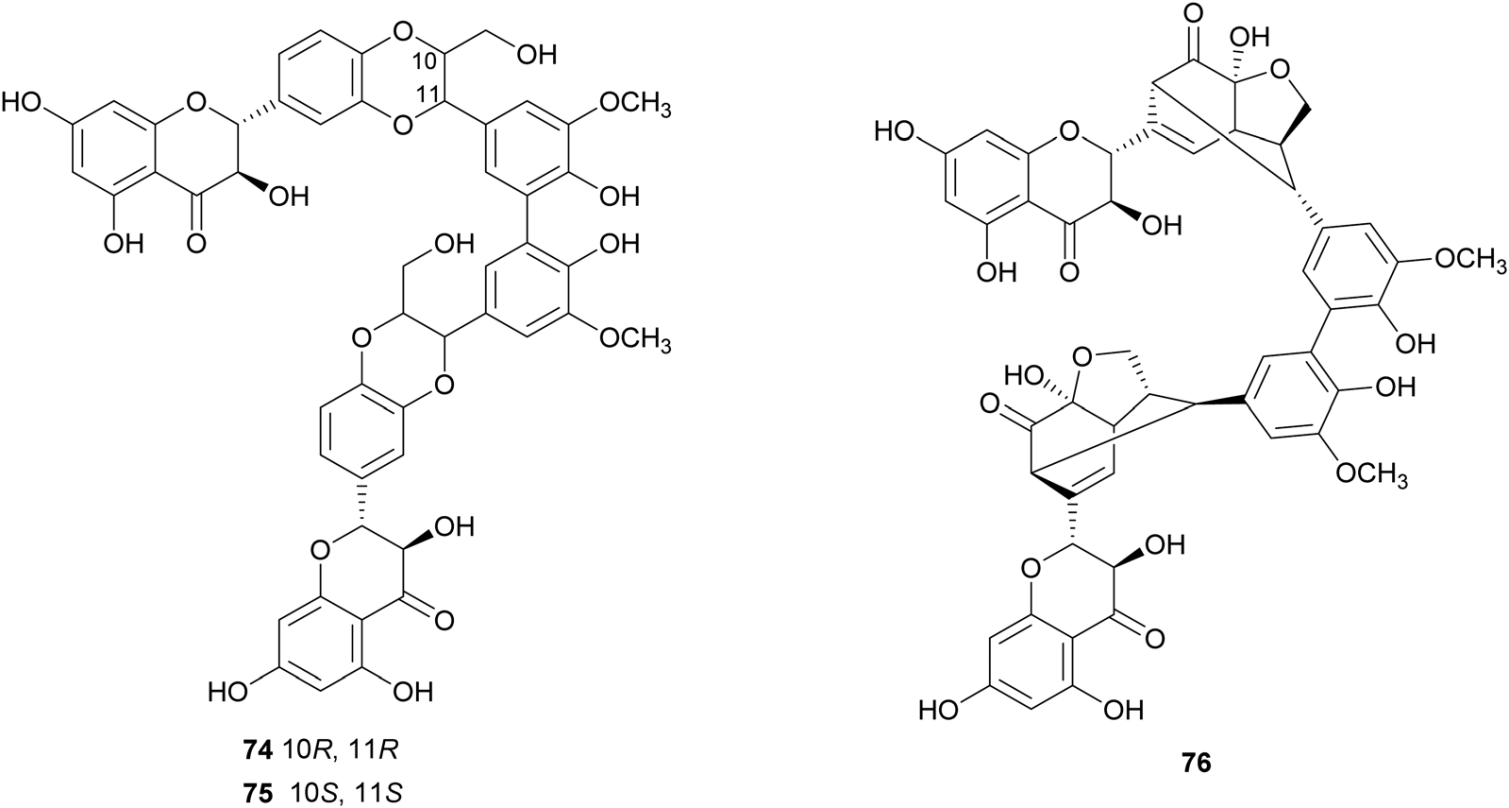


## 7. Biaryl compounds

**Scheme 16:**
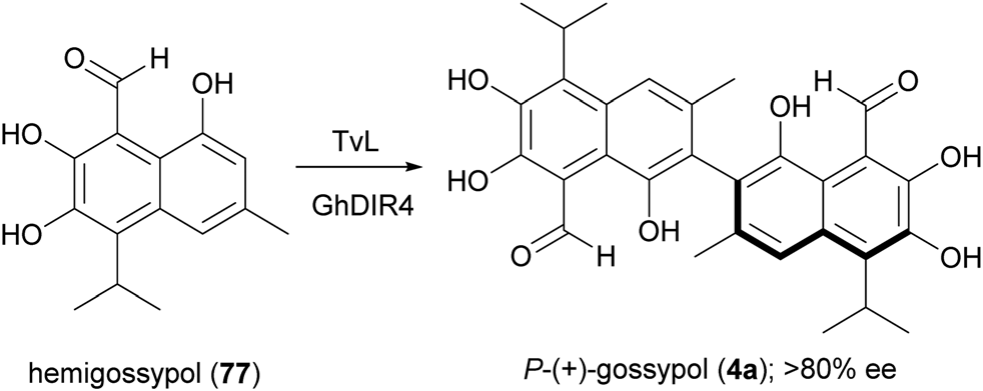
A combination of TvL and GhDIR4 catalyses the selective formation of *P*-(+)-gossypol.

A large variety of natural products is formed in Nature through a key oxidative step generating a radical at a phenol or at an aromatic amino group; by radical coupling, a biaryl core is then assembled, giving rise to symmetric or asymmetric skeletons. Laccase-mediated biaryl formation is reputed to be involved in biosynthesis of ellagitannins, aromatic polyketides, and other compounds.^131^ Considering the noticeable biological properties of some of these dimeric molecules,^29^ a number of biomimetic syntheses of biaryl compounds have been carried out, notwithstanding the inherent difficulties of this process, regarding regioselectivity, high steric repulsion and stereochemical outcome due to axial chirality in compounds with configurationally stable axes, such as gossypol (4). This is a defense compound of cotton plants (*Gossypium* spp.) and is endowed with multiple biological activities; it was initially used as a male contraceptive, but subsequently was found to be toxic for humans and non-ruminant animals, causing serious problems for the use of cottonseed oil for food and feed.^132^ It was then investigated as potential anticancer agent, and 4 together with its synthetic derivatives proved to have antitumor properties both *in vitro* and *in vivo*, also showing synergistic effects when used in association with other anticancer treatments.^133^ The biosynthesis of gossypol involves hemigossypol (77), a sesquiterpenoid; a peroxidase enzyme generates radical intermediates which by oxidative coupling give rise to a couple of atropisomers, *P*-(+)-gossipol (4a) and *M*-(−)-gossipol (4b).^134^ It is worth mentioning here that the two enantiomers show different biological properties, and 4b has a stronger cytotoxicity to tumour cells than 4a. Natural gossypol is a mixture of both enantiomers, with different ratio in different parts of cotton plant, although with a predominance of the dextrorotatory enantiomer (4a). It has been suggested that the stereocontrol in gossypol biosynthesis is due to a DIR. In an elegant work aimed to identify the factors orienting (+)-gossypol formation,^135^ the GhDIR4, was found to confer atroposelectivity to the oxidative coupling of hemigossypol in presence of TvL, thus allowing to obtain 4a with more than 80% e.e. (Scheme 16). This finding is notable considering that most cotton plants produces (+)-gossypol and (−)-gossypol at a 3 : 2 ratio, with very rare exception.

Other works focused on laccase-mediated dimerization of simple phenol derivatives, such as syringol, known as an antioxidant principle found in wood smoke and formed during thermal degradation of hardwood constituents.^136^ Employing a TpL fungal strain, syringol was oxidized in monophasic and biphasic system solvents and afforded the symmetrical C–C linked dimer 3,3′-5,5′-tetramethoxybiphenyl-4,4′-diol (78) with 20.9% yield. 78 showed antioxidant capacity (DPPH, FRAP) approximately double than that of syringol.^137^ In a later work, employing the laccase from *Botryosphaeria rhodina*, a selective formation of three different dimers was observed, and at pH 6.5 the dimer 78 was obtained as the only product. Experimental parameters were optimized, and the best reaction conditions afforded 78 with 12% yield.^138^ This dimer was evaluated as antioxidant to stabilize biodiesel, and proved to have antioxidant activity comparable to the commercial antioxidant butyl hydroxytoluene.

TvL was also employed to obtain nitrogenated dimeric compounds in good yields by homo and hetero-coupling.^139^ An oxidative coupling of *p*-chloroaniline catalysed by TvL in a biphasic system containing acetate buffer (50 mM, pH 4.5) and ethyl acetate : acetone (1 : 0.1) for 15 h at 22 °C, afforded 4,4′-biphenyl diamine (79) with 37%. 79 was a mild antifungal agent towards *Botrytis cinerea* and showed good antioxidant activity in the ORAC-PGR assay.^139^
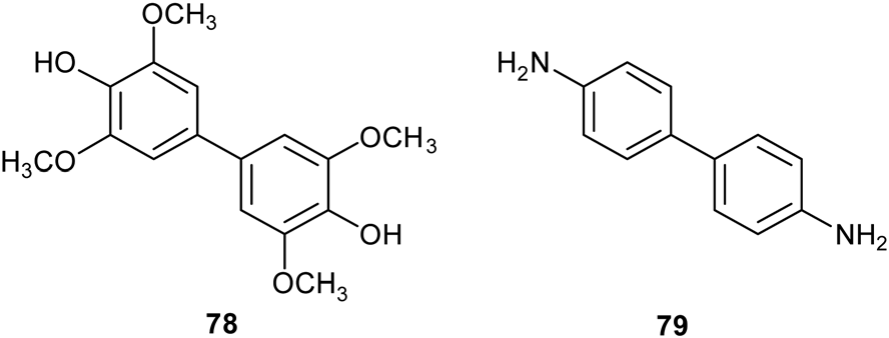


Lignin is one of the most abundant renewable biopolymers, constituting the lignocellulosic biomass. Thus, there is a rising interest toward the valorisation of lignin through degradation into smaller aromatic compounds employable as synthetic building blocks to obtain biologically active fine chemicals.^46^ Often these oxidative degradation reactions occur in the presence of mediators involving low molecular weight organic compounds,^140^ such those discussed in Section 2 (ABTS, TEMPO), in addition to natural products such as vanillin, and syringaldehyde. Within this frame, some vanillin-related compounds were tested as potential mediators. Firstly, the compounds were employed as substrates in laccase-mediated reactions affording various nitrogenated biaryl products.^141^ In particular, TvL and *Myceliophthora thermophila* laccase (MtL) catalyse the formation of five new biphenyls (80–84) with excellent conversion (from 72 to 99% yields), suggesting that vanillin-related compounds can be employed as mediators in LMS. Subsequently, some of them were used as mediators in presence of laccase for the oxidative coupling of lignin model compounds giving higher conversion yield (over 70%) respect to the reaction carried out in presence on natural mediators like vanillin (4%).^18^
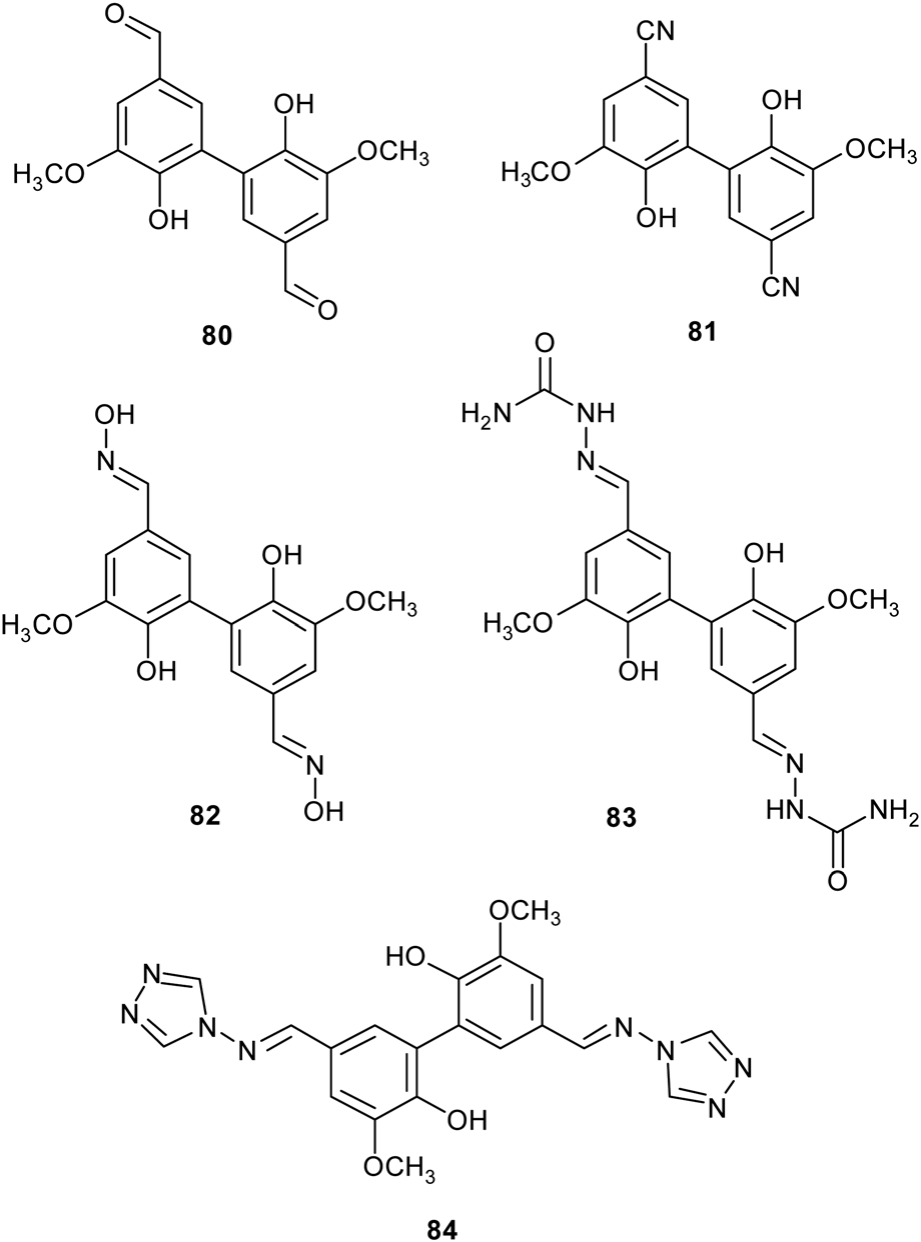


## 8. Miscellaneous compounds

**Scheme 17:**
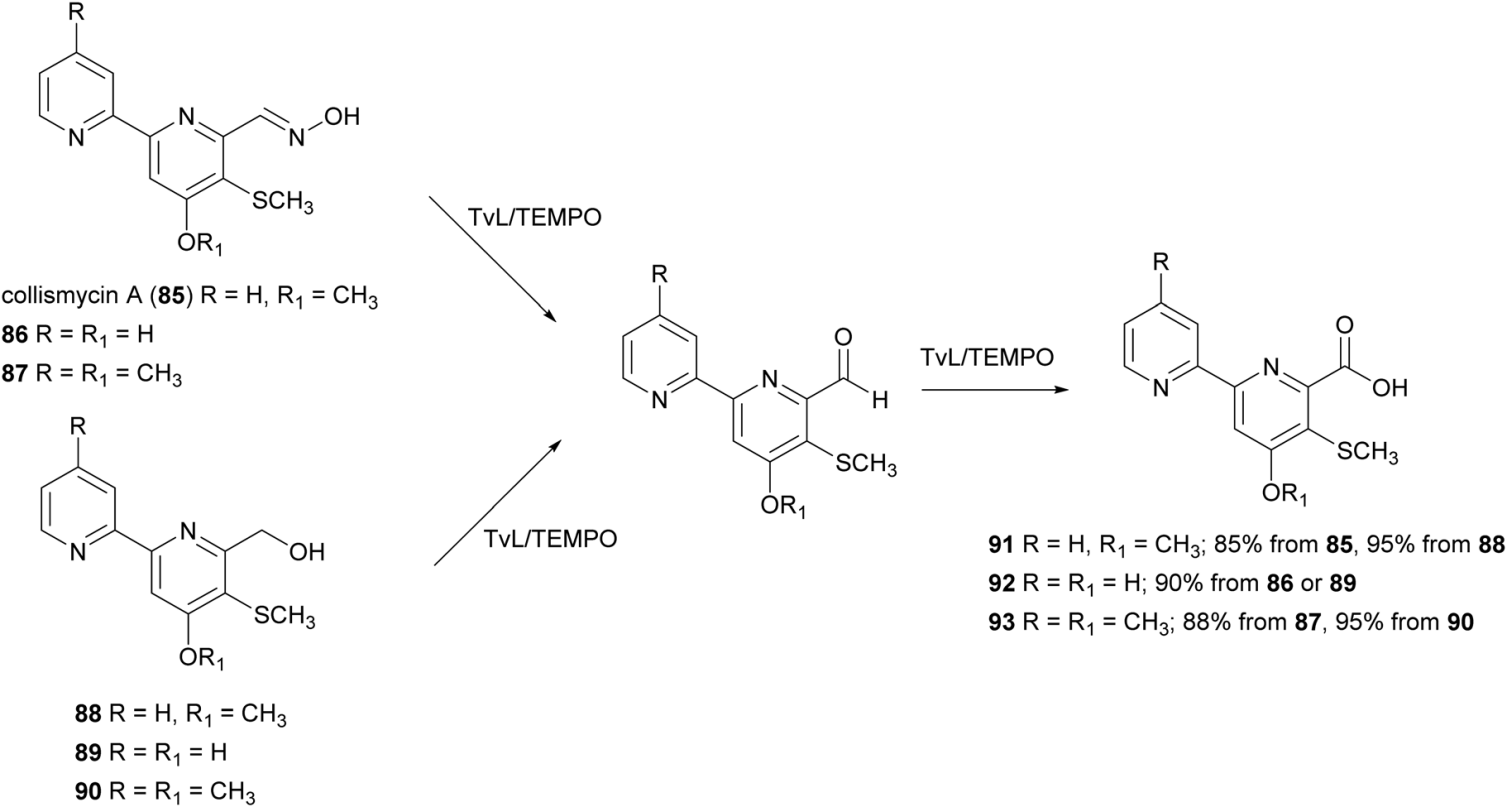
TvL/TEMPO-mediated synthesis of collismycin analogues 91–93.^144^

Collismycins are dipyridil-based bacterial metabolites displaying a wide range of biological properties, including cytotoxic, antifungal and antibacterial activity.^142,143^ One of the members of this family, collismycin A (85), is also a potent anti-inflammatory agent known for dexamethasone–glucocorticoid receptor-binding properties.^144^ An eco-friendly oxidation of 85 and other analogues (86–90) was carried out in water: acetonitrile (1 : 0.1) with 85–90 (0.04 mmol) in presence of TvL (1.84 U) and TEMPO (15% mol) and the carboxylic acids 91–93 were obtained with high yields ranging from 85 to 95%. According to this work, the conversion of aldoximes 85–87 into 91–93 occurred into two steps: (i) the oxime disproportionation into the corresponding aldehyde and hydroxylamine in the presence of mild acid conditions and laccase-TEMPO system; (ii) the conversion of aldehyde into carboxyl acids. Deconvolution experiments suggested that TEMPO transforms the oxime into a radical compound stabilised by the 2,2′-bipyridyl moiety, thus avoiding coupling reactions and enabling the attack of a water molecule after some evolution, to give, after re-oxidation, the carboxylic acid (Scheme 17).^144^

Ergot alkaloids are natural products with potent bioactivity, well-known in ancient times as poisoning agents; however, they also possess pharmacologically relevant properties and nowadays they are widely employed to treat various diseases, such as hypertension, senile cerebral circulatory insufficiency, migraine, post-partum bleeding and many others.^145^ Laccase-catalysed oxidation has been exploited to synthesize analogues of the ergot alkaloid *trans*-dihydrolysergol (94). For example, a reaction performed in TvL–TEMPO system in acetate buffer (20 mM, pH 3.5) allowed the stereoselective introduction of a hydroxyl group at the C-4 position of the ergoline skeleton (95) with 34.5% yield.^146^
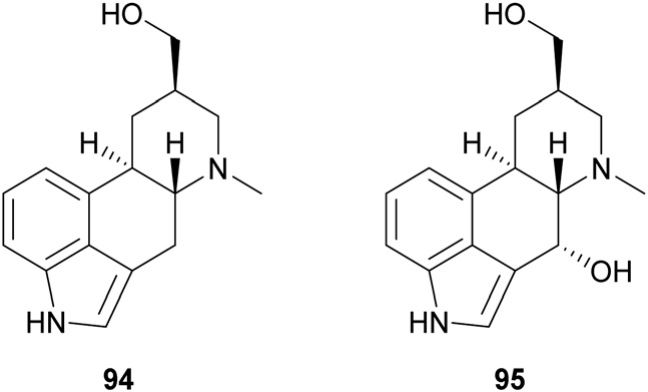


**Scheme 18:**
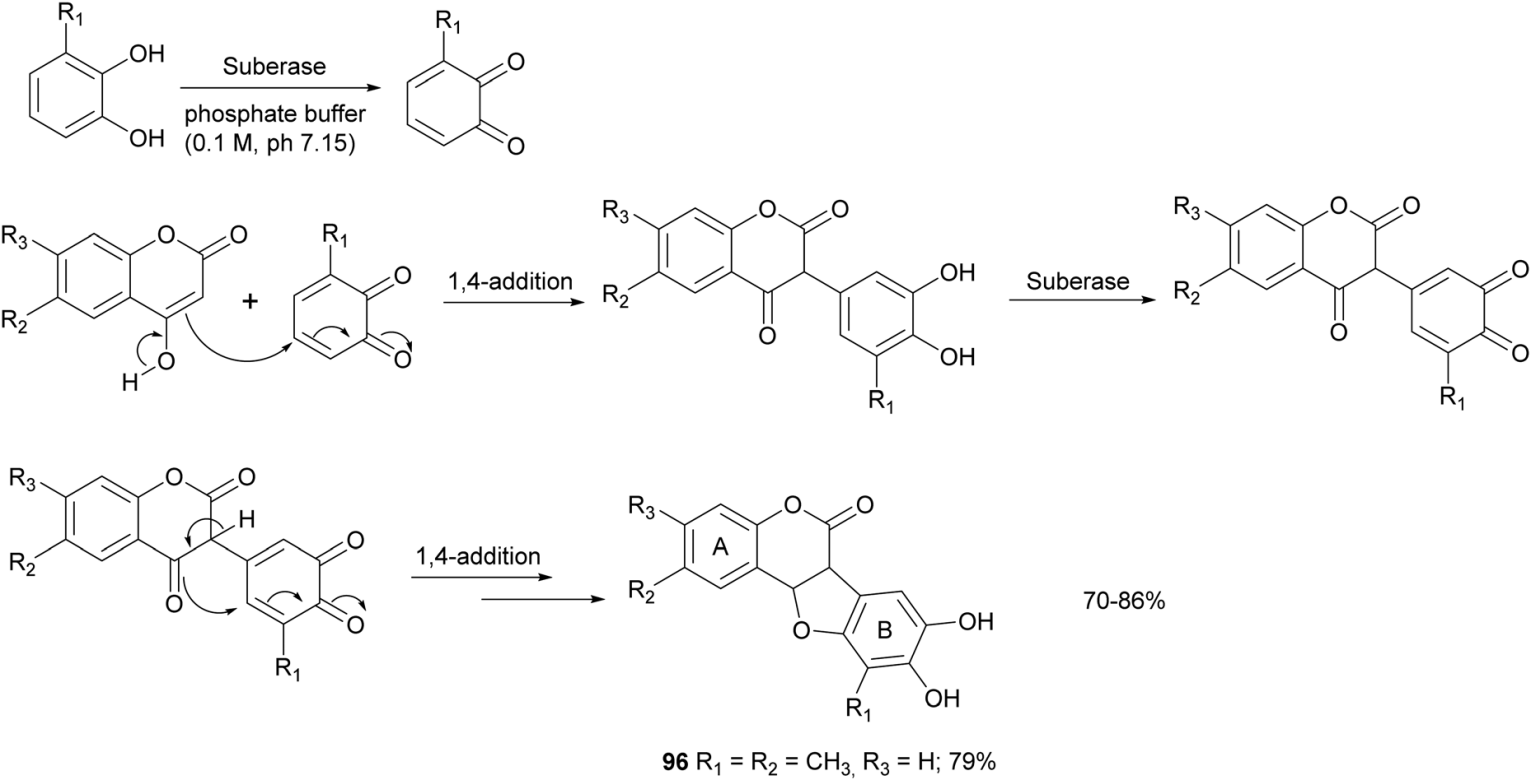
One-pot synthesis of coumestans mediated by laccase.

Some syntheses with a laccase-catalysed step have afforded bioinspired compounds with promising antitumor properties. In particular, fifteen coumestans were obtained employing catechols and coumarins as reagents in the presence of MtL commercialised as Suberase® (Scheme 18).^26^ The enzyme was added to a solution of substrate in phosphate buffer (0.10 M, pH 7.15) under air at rt. Laccase was added after 2, 18 and 20 h. The products, obtained in 70–86% yield and in high purity as determined by UPLC, exhibited weak or moderate antitumor activity towards MCF7 (breast), HeLa (cervical), UACC62 (melanoma), TK10 (renal) cell lines. The coumestan 96, displaying promising antitumor activity against breast MCF7 and melanoma UACC62 cell lines, bears a methyl group on rings A and B. In contrast, coumestans bearing chloro or fluoro substituents did not exert any significant anticancer activity.

**Scheme 19:**
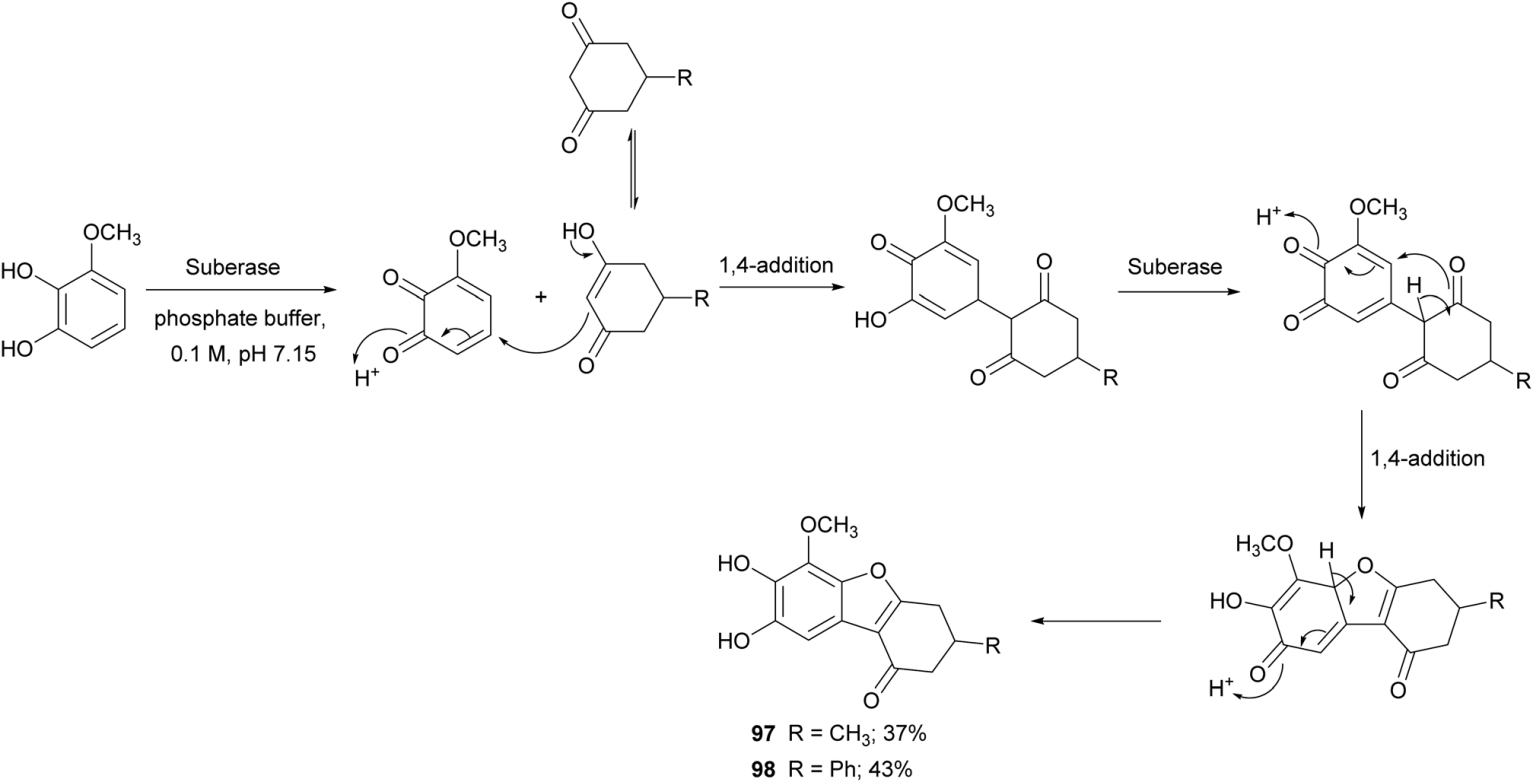
Synthesis mediated by a commercial laccase (Suberase®) of 5,6-dihydroxylated benzo[*b*]furans.

Benzofurans are widely distributed in plants kingdom and marine sources. These kinds of naturally occurring compounds have attracted the attention of synthetic organic chemists, due to their interesting biological and pharmacological activities. Several natural products bearing benzofuran scaffold are reported as antimicrobial, anti-inflammatory, antidiabetic and antitumor agents.^147^ Different synthetic approaches have been employed to synthesize natural and natural derived benzofurans. Among these, Suberase® has been employed to obtain hydroxylated benzo[*b*]furans starting from different catechols, thus affording 5,6-dihydroxylated benzo[*b*]furans with good yields (Scheme 19).^28^ The synthetized compounds proved to be potent and effective cytostatic agents against UACC62 melanoma cells and in particular compounds 97 and 98 had better antitumor activity than etoposide based on the growth inhibition data reported as GI_50_ values.

**Scheme 20:**
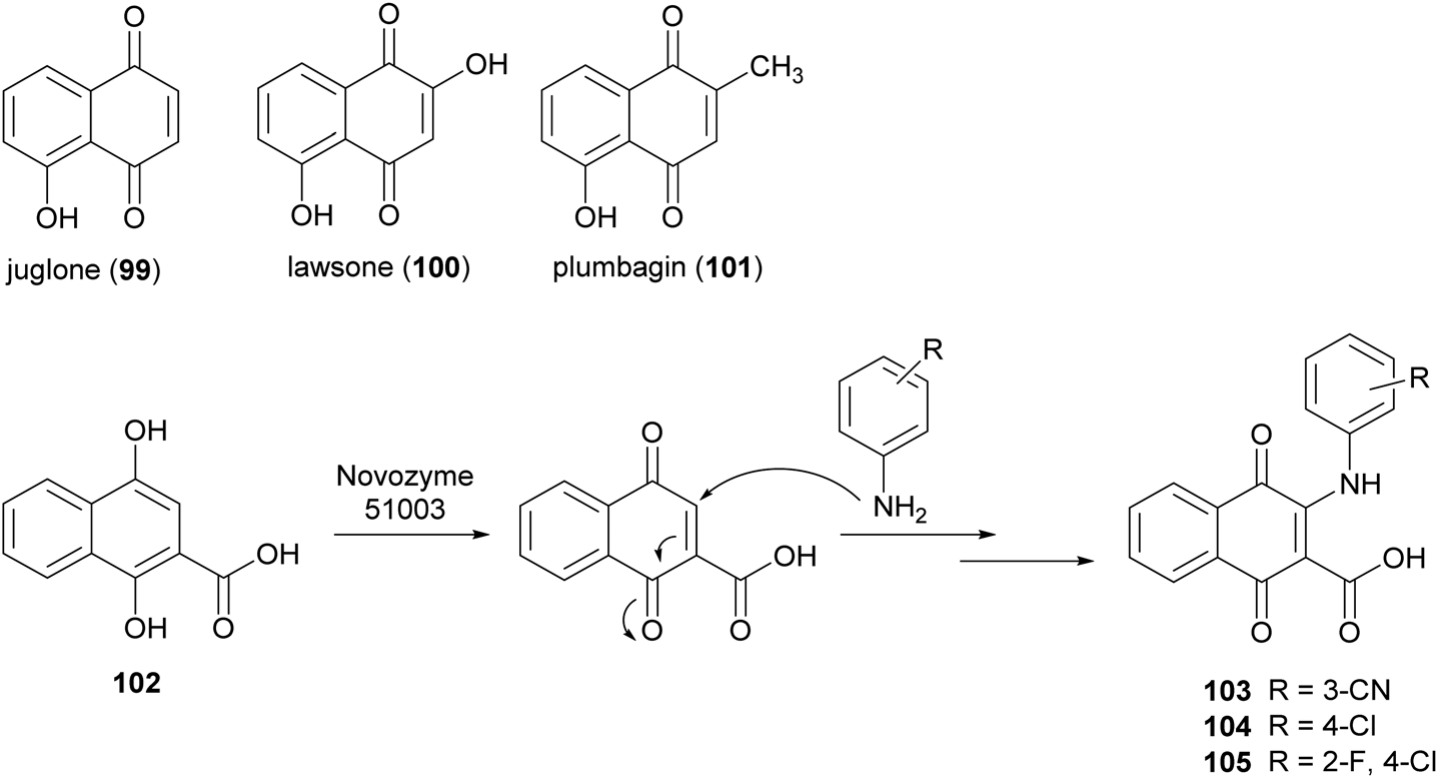
Synthesis mediated by laccase of aminonaphthoquinones.

Naphthoquinones are secondary metabolites as they are found in bacteria, fungi and higher plants. Juglone (99), lawsone (100), and plumbagin (101) are the most widespread products. Naphthoquinones display very significant pharmacological properties including antibacterial, antifungal, antiviral, insecticidal, anti-inflammatory, and anticancer activities.^148^ These properties are principally attributed to the oxidant characteristics of the naphthoquinones, which allow the generation of dianions or semiquinone radicals. Namely, electron transfer processes of quinones involve one or two-electrons and the properties of the reactive intermediates (either dianion or semiquinone), are related to their final biological activity.^149^ Examples include the involvement of quinones in allergy reactions, in energy conserving systems or as potent antitumor promotors. Two major mechanisms have been proposed for explaining the cytotoxic action of quinones in a variety of cell systems: (i) they act as redox-cycling agents, where they are readily reduced to semiquinone radical and reoxidized under physiological conditions, thus generating superoxide anion and H_2_O_2_; (ii) quinones are potent electrophiles, capable of reacting with the thiol groups of proteins as well as glutathione.^150^ The addition/substitution on naphthoquinone moiety with atoms or groups such as fluorine, oxygen, or amine can modulate redox properties, decreasing the toxicity levels and maintaining/potentiating the biological effect. In this context, Novozyme 51003® (a MtL expressed in genetically modified *Aspergillus* sp.) has been employed to synthesize new aminonaphthoquinones from 1,4-dihydroxy 2-naphthoic acid (102) with primary aromatic amines (Scheme 20) in DMF sodium phosphate buffer (10 mM M, pH 6.0) for 48 h.^27^ Among the naphthoquinone derivatives, compounds 103–105, obtained with 25–33% yields showed potent total cell growth inhibition (TGI) activity against melanoma with cytostatic effects 7- to 8-fold better than that etoposide.

In another work, the same commercial laccase Novozyme 51003® and TvL have been exploited for the synthesis of novel β-lactam antibiotics. The product pattern of the reactions achieved with both laccases were comparable, but the ratio of products formation differs according to the type of laccase. For preparative scale reactions, amino-β-lactams (10 mM) and 2,5-dihydroxyphenylacetic acid (3 mM) were incubated with Novozyme 51003® (final activity 800 nmol mL^−1^ min^−1^ in reaction mixture) in citrate phosphate buffer (pH 7.0) for 4 h.^87^ The compounds obtained were evaluated for their activities against Gram-positive bacterial strains, such as methicillin-resistant *Staphylococcus aureus* and vancomycin-resistant enterococci. Among the compounds tested, those bearing *para*-quinoid structures (106–109), obtained with 49.7–60.5% yield, showed more potent activity in protecting mice against infection with *S. aureus*.
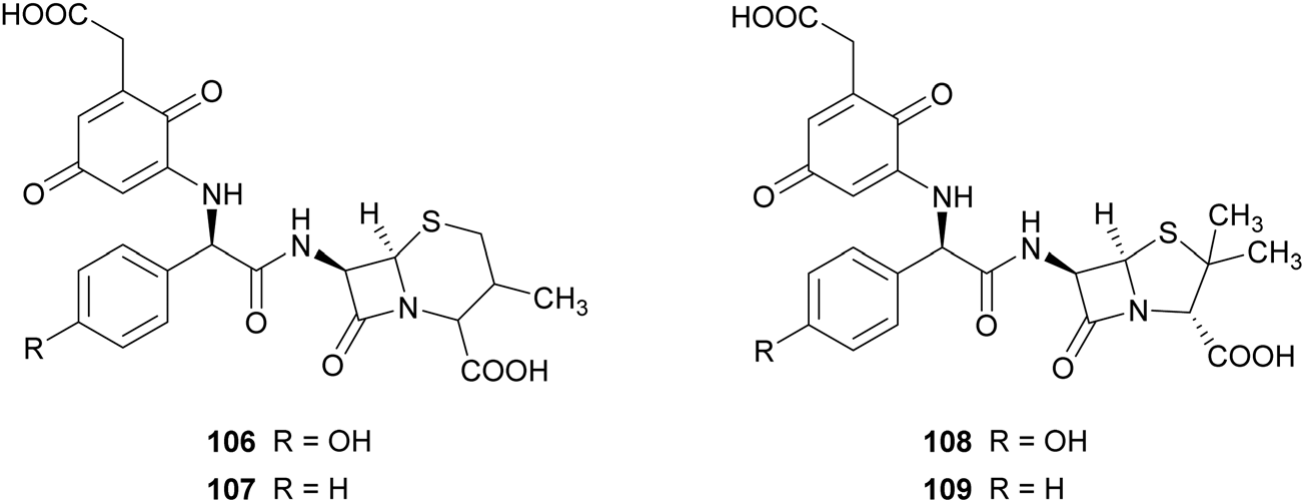


Phenazines, phenoxazines, and phenothiazines structures are often found in natural products and are known for their anticancer and antimicrobial activities.^151^ Examples of natural phenazines are pyocyanin (110) produced by *Pseudomonas aeruginosa* strains; cinnabarin (111) and cinnabarinic acid (112) are orange-red pigments of the phenoxazinone group obtained by fungi of the genus *Pycnoporus* (Polyporaceae).
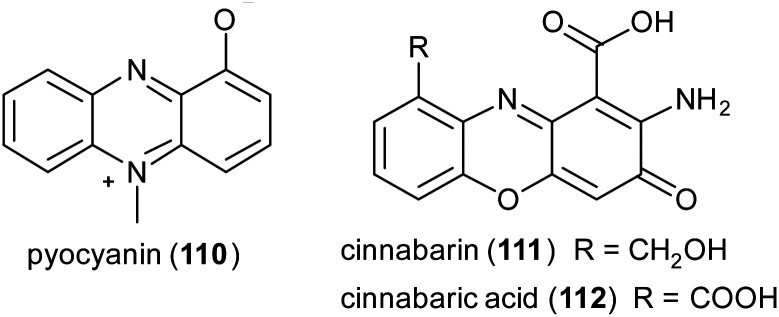


**Scheme 21:**
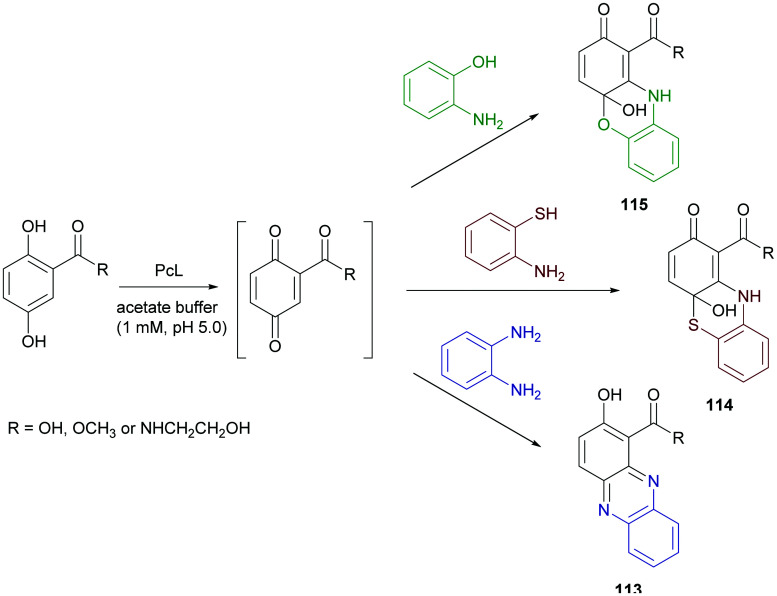
Laccase-catalysed synthesis of phenazines, phenathiazines and phenoxazines.

Phenazine derivatives hold a prominent position in medicinal chemistry owing to their wide spectrum of pharmacological properties. Synthetic analogues have been obtained and some of these compounds have shown activity similar to or even better than those of natural leads. In a recent work PcL in sodium acetate buffer (1 mM, pH 5.0) was employed to obtain phenazine (113), phenathiazine (114) and phenoxazine (115) analogues starting from 2,5-dihydroxy benzoic acid derivatives and *ortho*-phenylenediamine, 2-amino-thiophenol or 2-aminophenol, respectively (Scheme 21) with yields up to 29%.^86^

## 9. Natural polymers modification

Laccase-mediated modification and/or grafting on polymeric materials has gained considerable attention as an environmentally benign method to covalently modify polymers in order to obtain materials with new or improved properties/applications. In this context, an increasing number of works have been devoted to modifications of natural polymers mediated by laccases.^152^ Wood lignocellulose was subjected to laccase-assisted treatments to improve chemical or mechanical stability as well as to obtain biomaterials with antimicrobial and/or antioxidant activity. The modification of polysaccharides has furnished functional materials such as antibacterial and biodegradable plastics with promising biotechnological applications.

### 9.1 Lignin modification

Lignin is a readily available natural polymer with a complex cross-linked three-dimensional network structure. This natural polymer has a high carbon content, thermal stability, antioxidant properties, and biodegradability. Generally, native lignin is burned to provide steam and process heat for the pulp and paper mills. Its exploitation as a renewable material has been hindered by its complex structure, high molecular weight, high rigidity brittleness, and incompatibility with conventional polymers.^153^

**Scheme 22:**
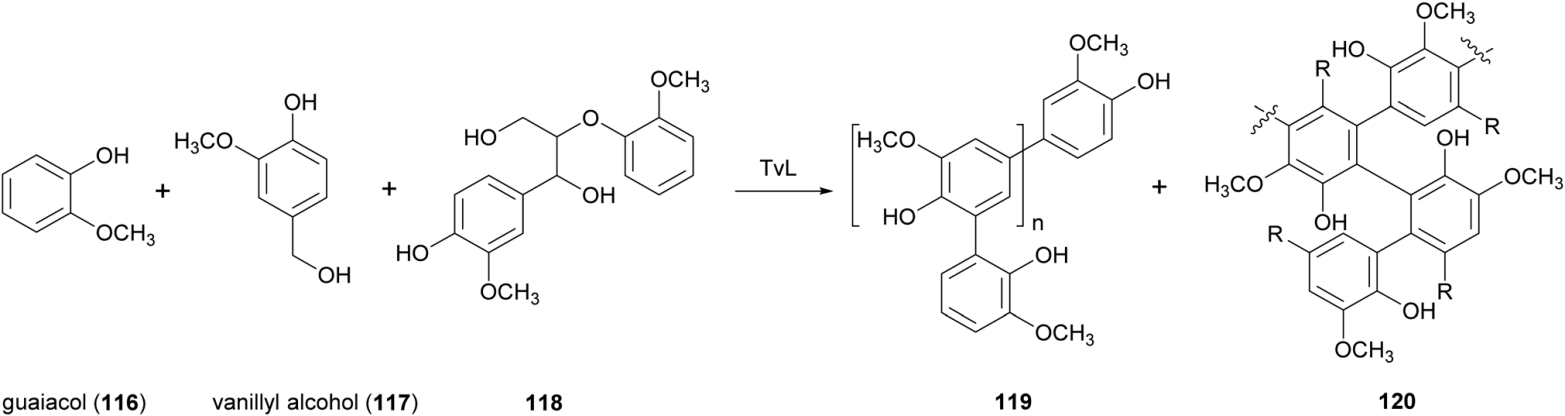
Lignin-like oligomers obtained by TvL-mediated synthesis.

Thus, different studies have been devoted to blend lignin with other polymers. For example, low molecular-weight lignin-like oligomers were obtained with two strategies: the first involving laccase-catalysed oxidative oligomerization of guaiacol (116) vanillyl alcohol (117) and dimeric lignin (118) in acetate buffer (pH 5.2) with 20% acetone or DMSO (Scheme 22), and the second based on oxidative depolymerization of commercially accessible kraft lignin by the employment chemical methods.^154^ The lignin-like oligomers with substructures 119 and 120 were incorporated into polyvinylchloride (PVC) films and were evaluated for UV-blocking characteristics. The results of this study suggested a possible use of this oligomeric materials as UV light blockers to protect polymers and plastics.

A laccase-assisted lignin oxidation/polymerization study was adopted to get insight on lignin structure–property performance correlation.^155^ This study employed two series of well-defined lignin fractions derived from birch and spruce alkaline lignin which were subjected to oxidation and polymerization *via* a new genetically evolved laccase (MetZyme) in an alkaline solution (pH 10) with a unique perspective for valorising lignin in biobased fibre products through green processing of solvent fractionation and enzymatic treatment.

When used alone, laccases can promote lignin polymerization with minor structural changes or depolymerisation, although the former process prevails. Lignosulfonates, obtained from sulphite process cooking *Eucalyptus globulus* wood, were subjected to oxidative polymerization in presence of laccase Novozyme® 51003. The product obtained with higher molecular weight up to 11-fold relative to starting material, has been designed as plasticizing additive in concrete formulations with improved performance.^156^

However, laccase redox potential is too low to fully oxidize the nonphenolic substructures of lignin, which account for up to 90% of the natural polymer. To overcome this recalcitrance, the use of mediators is frequently employed. For this reason, Hilgers *et al.* investigated in detail the reactivity of *p*-coumaroyl derivatives in lignin and model compounds in presence of laccase or a laccase-mediator system.^157^ The use of (HBT) and *N*-hydroxyacetanilide (HPI), both being –N–OH type mediators, were found to be incorporated to lignin in presence of TvL or *Pleurotus ostreatus* laccase.^158^ The grafting of these mediators may induce changes in the properties of lignin. Still, it may also serve as a potential pathway for controlling the modification of lignin with compounds possessing nucleophilic properties as both HBT and HPI are good leaving groups.

The synthesis of polymers based on modified lignin is often hindered by its intrinsic insolubility in common solvents and by the modest dispersion of high-molecular-weight native lignin into polymers due to the reduced interfacial binding. However, today's new perspectives are the development of new methods for lignin deconstruction and utilization for the production of value-added products. The efficient utilization of lignin requires its depolymerisation to low molecular weight phenolics and aromatics that can constitute building blocks for chemical syntheses of fine chemicals. A review article^159^ well describes the ability of laccase to degrade both phenolic and non-phenolic aromatic structures in lignin in conjunction with laccase mediators. Other examples report the depolymerisation of lignin by laccase in aqueous alkaline solution,^160^ by laccase-mediator systems in 1,4-dioxane/water,^161^ and an even greener procedure employing secretome from *Pleurotus eryngii*, with high laccase and peroxidase activity, in presence of the bacterial culture of *Pseudomonas putida*. The results indicated enhanced lignin depolymerization when the bacteria are present in combination with the secretome, reducing the lignin average molecular weight by 63–75%, and preventing repolymerization, thus demonstrating the concept called “microbial sink”.^153^

### 9.2 Cellulose modification

**Scheme 23:**
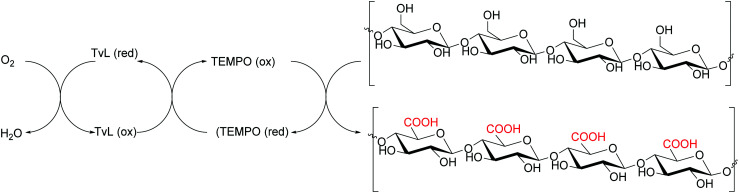
Preparation of oxidised cellulose with TvL/TEMPO.

Cellulose is one of the most abundant and renewable biopolymers existing as fine fibrils consisting of 30–40 cellulose molecules organized as fully extended chains. Cellulose nanofibers (CNFs) from plant cellulose microfibrils are a new class of materials interesting for their exceptionally high mechanical performance.^162^ Usually, CNFs are obtained by mechanical refining methods based on repeated high-pressure homogenization treatment of wood pulp/water slurries. However, such methods require high energy consumption. -To prepare CNFs with low energy consumption, some pretreatments of wood cellulose fibres, paper pulp or dissolving pulps have been developed. These pretreatment processes involve the use of TvL and TEMPO as mediator. This procedure has recently been employed to obtain oxidized celluloses with high C6-carboxylate contents from wood cellulose (hardwood bleached kraft pulp, HBKP, Scheme 23).^162^

In a similar manner, the oxidative treatment of bacterial cellulose (BC) with *Trametes villosa* laccase and TEMPO furnished a high functionalised BC (an increase up to 5 times in the concentration of carboxyl groups) and the paper produced with this cellulose showed enhanced mechanical properties and strong barrier function against water and greases compared to paper produced with non-oxidized BC. Moreover, the negative charge of the carboxyl groups of functionalized BC was used to generate silver nanoparticles (AgNPs), thus obtaining a BC paper and Ag composite with strong antimicrobial activity with high applicability in technological and biomedical uses.^163^

### 9.3 Chitosan modification

Chitosan is a natural-derived polymer with biocompatible, biodegradable, non-toxic features useful for applications in the biomedical, agricultural and functional food fields. This polysaccharide exhibits a strong metal ion chelating ability due to its nitrogen atoms, thus it can be used as antioxidant for metal ion deactivation. Unfortunately, it shows two main limitations to be considered a good antioxidant, such as poor solubility, due to the hydrogen bonds network and the lack of H-atom donors to act as an excellent chain-breaking antioxidant. Nevertheless, a chitosan-based biomaterial with enhanced antioxidant activity can be obtained through functionalization with natural polyphenols. Recent papers report the employment of laccase in the synthesis of biomaterials based on chitosan without harsh acidic solubilisation and organic solvent. For example, TvL was used to oxidize quercetin to the corresponding *o*-quinone which reacted with chitosan-free amino groups through Michael addition and Schiff base formation, thus obtaining new oligomeric products with structure like 121.^164^ The enzymatic reaction was conducted by dispersing and dissolving quercetin in phosphate buffer at 30 °C and pH 6.5. The scavenging activity of quercetin functionalized chitosan products towards ABTS^+^˙ was more than two times higher than that of native chitosan.
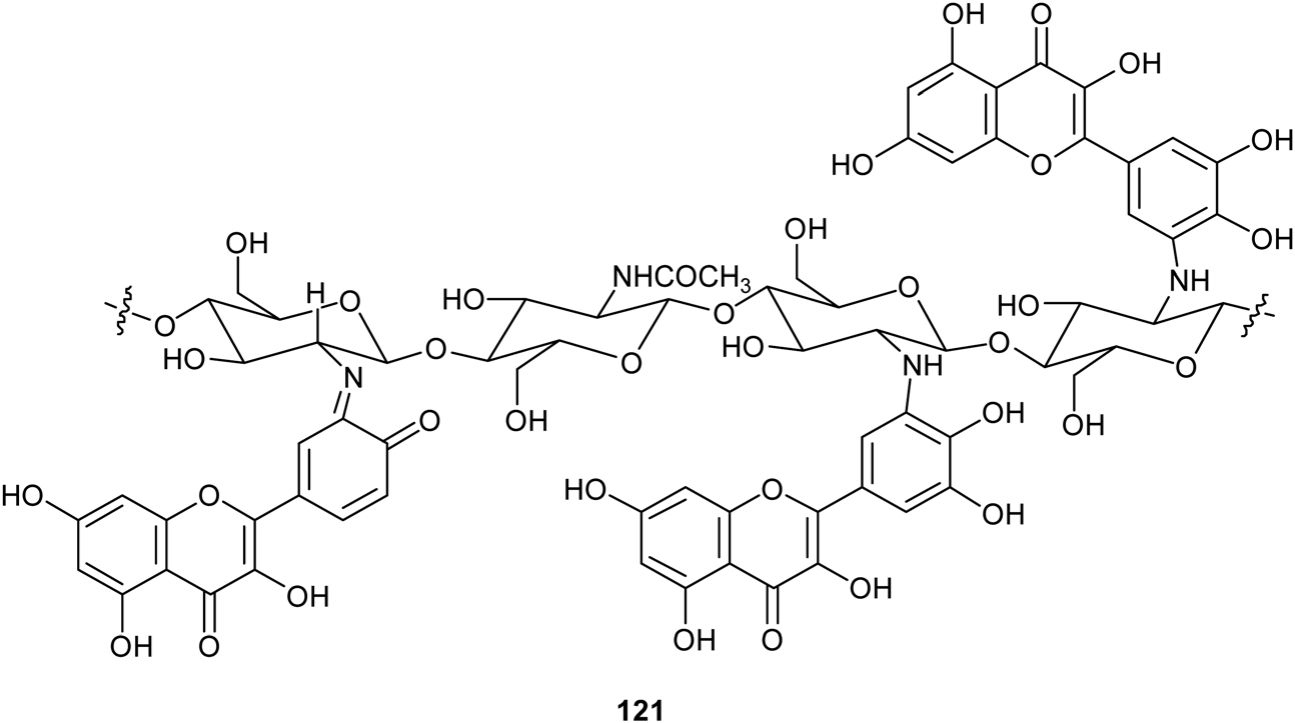


**Scheme 24:**
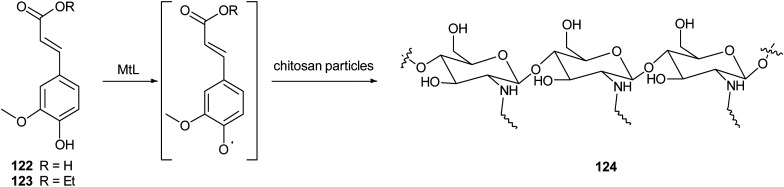
Schematic illustration of chitosan functionalization by laccase-catalysed oxidation of ferulic acid and ethyl ferulate under heterogeneous reaction conditions.

In another work, chitosan particles were functionalized by the oxidative products of ferulic acid (122) or its ethyl ester (123), in presence of MtL (Scheme 24).^165,166^ The enzymatic reaction was carried out under mild operational conditions (phosphate buffer at 30 °C and pH 7.5) with the solid chitosan particles suspended in the reaction medium. The reaction products of the oxidative step bound covalently to the free amino groups of chitosan, yielding products (exemplified as 124) with improved antioxidant properties, especially for ferulic acid–chitosan derivatives.

A recent application is the synthesis of chitosan-based hydrogels with potential application in wound dressings.^167^*O*-Carboxymethyl chitosan (CMCS) hydrogel-based derivatives were achieved by employing different natural cross-linkers (catechol, eugenol, caffeic acid, and sinapyl alcohol) using MtL. The highest degree of cross-linking (49.7%) was achieved with catechol. All the phenolic-CMCS hydrogels showed promising antioxidant activity up to 4-fold higher than in the absence of the phenolics. Furthermore, the hydrogels showed anti-inflammatory effects and no cytotoxicity for NIH 3T3 mouse fibroblast cell line.

An excellent example of the efficiency of laccase for textile processing is the employment of chitosan and catechin for binding onto a previous enzymatically oxidized linen surface (flax fibres) with an ascomycete laccase from MtL.^168^ The *o*-quinones formed by laccase oxidation promote the attachment of chitosan or/and catechin. The multifunctional material with both antioxidant and antibacterial properties was obtained with an acceptable level of durability in terms of end-user requirements.

Finally, chitosan has also been employed to enhance the stability and reusability of laccases through immobilization processes for several applications. TpL was entrapped onto chitosan beads using a glutaraldehyde cross-linker to improve the stability and recovery rate of the enzyme. This system was applied in the decolourization of various synthetic dyes.^169^ Other examples are employing immobilized laccase through chitosan nanoparticles on the glass beads for the decolourization of Congo red, a harmful industrial dye,^170^ and laccase encapsulation in chitosan nanoparticles to enhance the enzyme stability against microbial degradation in soil, compost, and wastewater.^171^

## 10. Concluding remarks

This review highlights the usefulness of laccases in organic synthesis of bioactive compounds. The reviewed recent literature reports many examples of laccase-mediated efficient syntheses of bioactive compounds, both natural or bioinspired. These include lignans, neolignans, dimeric stilbenoids, biflavonoids, biaryls, and others. Laccases or laccase-mediator systems provide environmentally friendly oxidation methods that can be used to replace chemical oxidants for a wide range of substrates.

All the examples of bioactive molecules of natural origin reported in this review are of terrestrial origin as the information and the number of examples of bioactive dimeric compounds of marine origin is very limited, though the biodiversity is greater in the oceans than on land. The research about the use of marine natural products as pharmaceutical agents suffers from many drawbacks like the lack of ethno-medical history, the difficulties involved in the collection and/or cultivation of marine organisms, as well as in their manipulation. However, in the next future, bioactive dehydrotyrosyl and dehydrodopyl compounds widespread among the marine invertebrates, as well as phlorotannins, phenolic compounds abundant in algae, could represent new natural substrates for the discovery of further laccase-mediated products with potential bioactivity for a variety of applications.

Many studies on laccase-mediated reactions have increased the understanding of reaction mechanisms involved and desired reaction conditions to yield a given product. Most of the reactions here reported employs enzymes of fungal origin (TvL or MtL). However, the definition of a specific laccase for a determined reaction has to take into consideration several parameters such as the substrate, pH, temperature, solvent and even the cost related to the consequent application. Other recent studies have focused their efforts to transfer this know-how from bench scale to industrial processes considering the need in the development of green chemistry solutions for pharmaceuticals and other industrial applications. In this context the reduction of cost for laccases production is the main issue in the feasible applications at an industrial level to be developed in the next future.

The recent results of the medium engineering and immobilization of laccases, thus improving the substrate scope and the enzyme efficiency, stability and recycling, suggest that in the next few years laccases will be used in in the generation of diverse compounds with different biological functions and for a variety of applications. Although these aspects have been extensively studied for the environmentally benign degradation of lignocellulosic biomass to afford high-value small products, not much is reported on the use of immobilized laccases or of deep eutectic solvents in the biocatalytic synthesis of fine chemicals. In this frame, it could be very advantageous to deepen the studies on eutectic solvents based on natural amino acids for the stereoselective synthesis of already known or new bioactive molecules for pharmaceutical applications.

Finally, though immobilization studies involving laccases have shown remarkable enzyme stability by retaining starting activity, very often the performances have been tested on model substrates or chemical mediators. The next decade could be devoted to the development and fine-tuning of the reaction conditions to afford fine chemicals, including pharmaceuticals.

## Conflicts of interest

There are no conflicts to declare.

## Supplementary Material
